# 3D-Printed GelMA Microneedles with Multilayer Therapeutic Properties for Diabetic Wound-Relevant Drug Delivery

**DOI:** 10.1021/acsomega.6c06574

**Published:** 2026-10-15

**Authors:** Shhd Saraj, Kaan Danis, Selcen Ari Yuka, Pelin Pelit Arayici, Sule Ilgar, Meryem Akkurt Yıldırım, Elif Kaya, Elif Ilhan, Gulgun Tınaz, Azime Erarslan, Oguzhan Gunduz, Cem Bulent Ustundag

**Affiliations:** † Department of Bioengineering, Faculty of Chemistry and Metallurgy, 52999 Yildiz Technical University, Istanbul 34220, Türkiye; ‡ Health Biotechnology Center for Excellence Joint Practice and Research (SABIOTEK), Yildiz Technical University, Istanbul 34220, Türkiye; § Institute of Health Sciences, Faculty of Pharmacy, Department of Biochemistry, 370018 Marmara University, Istanbul 34854, Turkey; ∥ Department of Basic Pharmaceutical Sciences, Faculty of Pharmacy, Marmara University, Istanbul 34668, Türkiye; ⊥ Nanotechnology and Biomaterials Research Group, Department of Metallurgical and Materials Engineering, Faculty of Technology, Marmara University, Istanbul 34840, Turkey

## Abstract

Type 2 diabetes mellitus (T2DM) is a chronic disease characterized by impaired insulin secretion, reduced insulin sensitivity, and hyperglycemia. Diabetic wounds are among the major complications of T2DM and can lead to chronic wounds due to challenges in the normal healing process. In this study, a three-layered tissue scaffold incorporating microneedles (MNs) was developed for diabetic wound management. The scaffold was fabricated using digital light processing (DLP) for the GelMA-based layers and electrohydrodynamic atomization (EHDA) for the surface coating, combining antibacterial, metabolic, and antioxidant functions within a single platform. The first two layers were designed using gelatin methacrylate (GelMA) and produced via DLP. The first layer was loaded with the antibiotic amoxicillin to prevent infection at the scaffold application site. In the second layer, MNs containing metformin were designed. For the outermost layer, polylactic acid (PLA) microparticles containing antioxidant and antibacterial salicylic acid (SA) were produced via the EHDA method and coated onto the scaffold surface. This three-layered scaffold was characterized by SEM, FT-IR, DSC, and mechanical, swelling, degradation, and penetration analyses. *In vitro* studies of single-drug-loaded model formulations demonstrated biphasic drug-release behavior over 72 h, effective antibacterial activity against *E. coli* and *S. aureus*, and favorable cytocompatibility. Oxidative-stress assays showed lower Total Oxidant Status (TOS) levels and higher Total Antioxidant Status (TAS) values in the drug-loaded formulations, while the scratch assay demonstrated increased wound-gap closure under hyperglycemic culture conditions. Overall, the multilayer microneedle platform exhibited antibacterial activity, with inhibition zones of up to 23 mm, and favorable cytocompatibility, with cell viability remaining above 77% across all formulations. These findings provide *in vitro* proof-of-concept evidence for the multifunctional design of the platform but do not demonstrate wound-healing efficacy in diabetic tissue.

## Graphical abstract

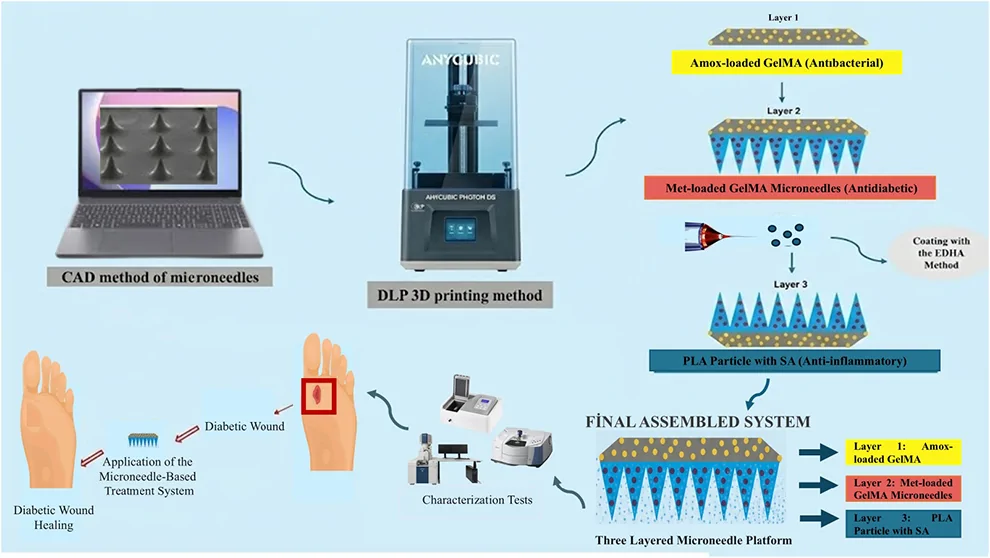

## 1 Introduction

Diabetes Mellitus (DM), characterized by high blood sugar levels, is a serious chronic metabolic disease that is also frequently accompanied by hyperglycemia and various complications (including cardiovascular and chronic kidney diseases).^1,2^ DM is becoming increasingly prevalent, according to the 2025 International Diabetes Federation Diabetes Atlas, approximately 589 million adults aged 20–79 years were living with diabetes worldwide in 2024, and this number is projected to increase to 853 million by 2050.^3^ Among the two types of this metabolic disease defined as type 1 diabetes (T1DM) and type 2 diabetes (T2DM), T2DM is the most prevalent, and patients often suffer from wounds on the skin, most commonly on the lower extremities (especially the feet).^4^ High blood sugar can compromise the body’s immune system, which can make diabetics susceptible to various types of infection. In addition, as a result of damage to the inner lining of the blood vessels, poor circulation cannot supply enough oxygen and nutrients to the injured area. As a result of disruptions in these essential biological processes, the healing process is significantly impaired, especially in the lower extremities, such as the feet.^5^

Normal wound healing is a multifaceted and dynamic process that results in the restoration of anatomical integrity with similar functions. While acute wounds usually heal normally without complications, DM patients often result in chronic damage with prolonged healing time of more than 12 weeks, often accompanied by an anomalous progression of healing stages.^6^ Among the many surgical and nonsurgical methods to treat chronic wounds, one of the most common methods is wound dressings that allow the wound area to be covered and promote the formation of new tissue and blood vessels.^7^ In recent years, microneedle (MN) designs comprising of submillimeter needles have been integrated into wound dressings to provide painless penetration of therapeutic molecules into multiple dermal layers. Considered as minimally invasive medical interventions, these systems have been proposed for the treatment of various diseases due to their effective delivery of therapeutic molecules.^8^ Depending on the material and design, fabrication of MNs can be accomplished by various methods such as lithography/machining with selective etching, laser ablation, micromilling, soft lithography, microinjection molding, hot embossing and 3D printing.^9^ Of these, 3D printing technology is a manufacturing technique that has been increasingly used in recent years in the development of patient-specific adaptive hard and soft tissue analogs.^10^ 3D printing systems, Digital Light Processing (DLP) technology allows the formation of structures by processing and hardening the relevant photopolymer layers with light. This method allows the production of special designs such as GelMA MNs.

GelMA, which is obtained by modification of natural gelatin and has the ability to cross-link by exposure to UV or visible light, possesses various natural motifs that can stimulate cell attachment and interactions of matrix metalloproteinases.^11^ Thus, it has the potential to fulfill its natural ECM function and has been frequently used in tissue engineering applications in recent years.^12,13^ The development of fine-tuned applications during the synthesis of GelMA determines the degree of functionalization, which allows us to control the mechanical and physical properties of GelMA hydrogels.^14^ Several perspectives have been developed to utilize these superior properties of GelMA-based microneedle approaches in chronic wounds. For instance, GelMA/PEGDA (poly­(ethylene glycol) diacrylate) microneedle patches developed to provide controlled release of exosomes and tazarotene have been shown to promote cell migration and blood vessel formation in deep skin layers while maintaining biological activity. Experiments in a diabetic mouse model revealed that GelMA/PEGDA@T + exos (GelMA/poly­(ethylene glycol)) diacrylate (PEGDA) microneedle patches loaded with tazarotene and exosomes (GelMA/PEGDA@T + exos) microneedle patches offer a potential method for the treatment of diabetic wounds.^15^ For example, GelMA hydrogel was used as a base material in a dual-action microneedle approach with antibacterial and reactive oxygen species (ROS)-scavenging properties. Immobilization of antimicrobial peptide/MnO_2_ hybrid nanoparticles on GelMA hydrogel platform and its induction by photothermal stimuli resulted in peptide-mediated antibacterial and MnO_2_-mediated antioxidant activity in chronic wound site.^16^ While these recent studies demonstrate the potential of GelMA-based MN platforms, many more approaches are needed before their use in diabetic chronic wounds can be translated into clinical applications.

The three agents were selected to target distinct, established barriers to diabetic wound healing. Amoxicillin was incorporated into the base layer to reduce local bacterial burden, a major complicating factor in diabetic foot ulcers; this may also indirectly reduce infection-associated oxidative stress.^17^ Metformin’s systemic role in glycaemic control is well established, but it has also been reported to influence AMPK-related signaling in keratinocytes, a pathway associated with inflammatory responses and redox balance under hyperglycemic stress.^18–20^ This provides a distinct rationale for its local delivery via the microneedle layer rather than relying solely on systemic dosing. Salicylic acid was incorporated into the outer PLA coating for its combined keratolytic, antioxidant, and mild antibacterial properties. Its keratolytic action may help soften the hyperkeratotic and callused tissue that frequently surrounds diabetic foot ulcers,^21,22^ while its reported antioxidant activity may help reduce oxidative stress within the wound microenvironment.^23,24^ Together, the three agents were selected to address infection, local metabolic/cellular dysfunction, and oxidative stress as three interlinked barriers to healing.

Compared with previously reported GelMA-based microneedle platforms, which typically combine a single drug class with a biological or nanomaterial-based adjunct (e.g., exosomes, antimicrobial peptide (AMP)/MnO_2_ nanoparticles), the present system integrates three clinically established, low-cost small-molecule drugs, each assigned to a distinct spatial layer matched to a distinct wound-healing barrier (infection, local metabolic/migratory dysfunction, and oxidative stress/hyperkeratosis). It also combines two fabrication modalities, DLP vat photopolymerization and EHDA as given in Table 1.

**Table 1:** Comparison of the Present Platform with Related GelMA-Based Microneedle and Multilayer Wound-Delivery Systems

Platform	Fabrication	Active agent(s)	Functions targeted	Evidence level
This study	DLP (GelMA MN) + EHDA (PLA/SA coating)	Amoxicillin, Metformin and Salicylic acid, incorporated into 3 separated layers	Antibacterial activity, hyperglycemia-relevant cellular support, and antioxidant/keratolytic functions	*In vitro*
Yuan et al.,^15^	Molding	Exosomes + Tazarotene	Pro-angiogenic/pro-migratory + keratinocyte differentiation	*In vitro* + *in vivo* diabetic mouse model
Wang et al.,^16^	GelMA hydrogel MN (photothermal-triggered)	AMP/MnO_2_ hybrid nanoparticles	Antibacterial + ROS-scavenging	*In vitro* + *in vivo* *S. aureus*-infected rat wound model
Harmanci et al.,^10^	3D-printed PCL/PVA bi/trilayer sandwich scaffold (not a microneedle)	Metformin	Metformin delivery and wound-dressing-related physicochemical performance	*In vitro*

In this work, we developed a 3D-printed, multilayer GelMA-based microneedle platform specifically designed to address the complex biological and functional requirements of diabetic wound environments. The platform was engineered with three therapeutic layers: amoxicillin incorporated into the GelMA base to provide antibacterial protection, metformin embedded within the microneedle layer to provide localized metabolic modulation under diabetic wound-relevant conditions, and Polylactic acid (PLA) microparticles loaded with SA electrosprayed onto the surface to contribute to antioxidant and wound-relevant supportive properties. In the following sections, we present the characterization, mechanical performance, degradation behavior, drug release, antibacterial activity, cytocompatibility, and penetration efficiency of this multifunctional system, demonstrating its potential as a promising minimally invasive approach for diabetic wound treatment.

## 2 Materials and Methods

### 2.1 Materials

Gelatin (Type B, 230–250 g Bloom) from bovine skin was from Halavet (0797–21/Turkey). Methacrylic anhydride (MAA) (276685–100 mL), salicylic acid (SA) (MW: 138 g/mol), lithium phenyl-2,4,6-trimethyl-benzoyl phosphinate (LAP, MW: 294.21 g/mol, 95% purity), dialysis membrane (MWCO 12–14 kDa), amoxicillin and metformin were from Sigma-Aldrich. Sodium carbonate and sodium hydroxide was from Merck KGaA (Darmstadt, Germany). Sodium hydrogen carbonate (>99.7%) was from ISOLAB (Eschau, Germany). PLA in granule form was from Nature Works LLC (PLA 4060D).

### 2.2 GelMA Synthesis

Gelatin was dissolved in a 10% (w/v) bicarbonate buffer (1 L of distilled water containing 3.18 g sodium carbonate and 5.86 g sodium bicarbonate) at 60 °C under thorough mixing until complete dissolution. For each gram of gelatin, 0.2 mL of methacrylic anhydride (MAA) was added to the solution. The mixture was allowed to react for 4 h at 50 °C under constant stirring. After the reaction, the gelatin mixture was dialyzed against deionized water using a dialysis membrane for 5 days to remove unreacted methacrylic anhydride. The purified GelMA solution was lyophilized after filtration and stored at −20 °C until further use.^25^

### 2.3 Determination of Substitution Degree

The produced GelMA substitution degree was analyzed using ^1^H NMR spectroscopy (Bruker AVANCE). Using D_2_O solvent GelMA and gelatin were dissolved separately in 10 mg/mL concentrations. ^1^H NMR spectra was obtained in room conditions using 500 MHz frequency with the degree of substitution being calculated with the following eq ([Disp-formula eq1])

1 $$\mathrm{DS}=\left(1-\frac{GelMA\;ly\sin e\;peak\;area}{Gelatin\;ly\sin e\;peak\;area}\right)\times 100$$

In this study, the integral areas were determined based on the reference peak at ∼2.9 ppm which corresponds to the lysine methylene protons in gelatin and GelMA.^26^

### 2.4 Microneedle Design and Printing

Optimized GelMA MN parameters and drawing was continued from previous works.^12^ Briefly, computer-aided design (CAD) files of tapered microneedle arrays, with needle heights in micrometers, were prepared using SolidWorks 2020. Each microneedle was designed to be 2000 μm height and bonded to a solid 10 mm × 10 mm × 1 mm base containing a 6 × 6 array. The microneedle designs were then converted into.stl file format and sliced using the 3D bioprinting software Chitubox, with an exposure time of 70 s set during the slicing process. DLP-based 3D printer (Anycubic Photon D2, Anycubic Technology Co., Ltd., Shenzhen, China) was used which possess 405 nm light wavelength UV-LED projector.

DLP printing process prints layer by layer. The MN drawing has a total of 150 layers with each layer thickness being 0.02 mm. GelMA (20% w/v) in PBS with LAP at 2.5 mg/mL was used as the bioink to obtain these MNs. Following the protocol established for amoxicillin-loaded GelMA microneedles by our group.^27^ Amoxicillin and metformin were weighed and added directly to the drug-loaded GelMA bioinks, which were homogenized using a magnetic stirrer prior to printing. To produce the multi layered construct, the solution for the base layer (loaded with amoxicillin) was added to the vat and the printing process was started. After the completion of the 75th layer the device was paused, old vat was removed and new vat was added. The needle solution (loaded with metformin) was added to the new vat and printing was resumed. Following printing, microneedles were washed to remove uncured bioink; no additional postcuring step was performed.

### 2.5 Electrohydrodynamic Atomization (EHDA)

The EHDA process was performed according to previously reported methods with minor modifications.^28^ To obtain microsized particles, 0.05% (w/v) PLA was dissolved in chloroform, and salicylic acid was added at 4 wt % relative to PLA mass. The solution was then loaded into a 10 mL syringe, and electrosprayed under 20 kV with a 20 μL/min flow rate. The distance from the nozzle tip to the collector was optimized at 14 cm. The produced samples material compositions are given in Table 2.

**Table 2:** Details of the Produced MN Samples

Samples	GelMA Base Drug	GelMA needle Drug	PLA microparticles
GelMA-MN	-	-	-
A-M-GelMA-MN	5% (wt % of the polymer) Amoxicillin	1% (wt % of the polymer) Metformin	-
A-M-GelMA-MN@SA	5% (wt % of the polymer) Amoxicillin	1% (wt % of the polymer) Metformin	4% (wt % of the polymer) Salicylic acid

### 2.6 Chemical Characteristics of the Microneedle (FT-IR)

The molecular structure and chemical properties of the MNs produced were investigated using FT-IR (Fourier Transform Infrared) (PerkinElmer spectrum 100, USA) spectroscopy. Spectrum analysis was performed in the 400–4000 cm^–1^ scanning range and at 4 cm^–1^ resolution.

### 2.7 SEM Analysis

The surface morphology and size of the MNs produced and sprayed microparticles were examined using SEM (EVA MA 10, ZEISS, USA). Before imaging, the surface of the samples was coated with gold for 120 s with a sputter coating machine (Quorum SC7620, USA). Then the analysis was conducted. SEM images were acquired under an accelerating voltage of 10 kV at magnifications of 75×, 500×, and 2000×. Microneedle heights and microparticle diameters were measured using ImageJ software.

### 2.8 Thermal Characteristics of the Microneedle

The thermal behavior of the MNs was tested using a differential scanning calorimeter (DSC, Shimadzu, Japan), The MNs were examined in the temperature range of 25–300 °C at a heating rate of 10 °C/min under air conditions.

### 2.9 Mechanical Testing

The mechanical characteristics of the MNs were determined via compression tests. A compression testing apparatus (EZ-LX, Shimadzu Corporation, Japan) with a 5 kN ± 0.5% load cell at ambient temperature and a displacement resolution of 1 μm was used for the tests. Cylindrical hydrogels were prepared according to MN samples parameters (same amount of GelMA and drug loading, same DLP parameters) with a height of 2.5 mm and a diameter of 7.0 mm. Compression tests were conducted at a rate of 1 mm/min. Analysis was conducted in triplicate and standard deviation was given.^29^

### 2.10 Swelling and Degradation of the Microneedle

The MNs were prepared in ∼10 mg samples. These samples were kept in 2 mL PBS in a thermal shaker at 37 °C. Prior to addition into PBS, the initial weights of the MNs (*W*_0_) were measured using an analytical balance. The samples were then taken from PBS at 1, 5, 15, 30, 60, 90, 120, 150, 180 min time intervals, and their weight was measured (*W*_w_). After each measurement, the PBS solution was replaced with fresh PBS. The swelling value (*S*) was calculated using the eq ([Disp-formula eq2])^30^

2 $$S=\frac{W_{w}-W_{0}}{W_{0}}\times 100$$

For degradation, the microneedle samples’ initial weights (*W*_0_) were again weighed using an analytical balance. These samples were put in PBS (2 mL), removed from the PBS medium every 5 days (1, 5, 10, 15, 20, 25, 30 days) and dried at room temperature for 5 h, then their weights were calculated. The degradation value (*D*_i_) was calculated using the following eq ([Disp-formula eq3]) by using the sample weights (*W*_t_)^31^

3 $$D_{i}=\frac{W_{0}-W_{t}}{W_{0}}\times 100$$

These analyses were conducted in triplicates, and the mean standard deviation was given.

### 2.11 *In Vitro* Drug Release

Prior to evaluating release kinetics, the incorporation of the active agents within the microneedle sample were confirmed. Detailed quantitative methodology and results regarding the encapsulation efficiency (EE%) and actual drug loading (DL%) for amoxicillin, metformin, and salicylic acid are provided in the Supporting Information (Table S1).

The release behavior of amoxicillin, metformin, and SA from the MNs was evaluated under *in vitro* conditions. Calibration curves of the drugs were drawn. Amoxicillin, metformin and SA’s best absorbance values were determined as 228, 232, and 298 nm with the *r* ^2^ values found for each drug as 0.998, 0.9994, and 0.9993 respectively. To draw the cumulative release graph of the drugs from the MNs, 9 mg of the MN samples were prepared. Release studies were performed by incubating microneedle samples in 1 mL of PBS (pH 7.4) at 37 °C under continuous agitation at 200 rpm. At each predetermined time point, the entire 1 mL release medium was withdrawn for analysis and immediately replaced with 1 mL of fresh PBS, and sink conditions were maintained throughout the study. Due to the UV absorbance spectra’s of amoxicillin, metformin, and salicylic acid overlapping, release studies were conducted using single-drug-loaded model formulations corresponding to each layer. Therefore, the presented release profiles represent layer-specific release behavior rather than simultaneous quantification from the complete multilayer construct. Samples were analyzed within time intervals of 15 min, 30 min, 1, 2, 3, 4, 6, 12, 18, and 24 h and 2, 3 days using UV–vis spectrometer (Shimadzu, Japan). Every sample was done in thrice and its mean was taken as the final value.

To elucidate the drug release mechanisms from the respective GelMA and PLA matrices, the cumulative release data were fitted to various mathematical kinetic models (Zero-order, First-order, Higuchi, Korsmeyer–Peppas, and Hixson–Crowell). The detailed kinetic modeling and corresponding correlation coefficients (*R* ^2^) are provided in the Supporting Information (Figure S2, Table S2).

### 2.12 Antibacterial Activity of the Microneedle

The antibacterial activities of the samples were tested using the disc diffusion method specified by the Clinical Laboratory Standards Institute (CLSI). *Staphylococcus aureus* *ATCC 25923* (*S. aureus*) and *Escherichia coli* *ATCC 25922* (*E. coli*) bacteria were cultured on LB medium and incubated overnight at 37 °C. After the overnight bacterial cultures were adjusted to Mcfarland 0.5 density, they were spread on Mueller–Hinton Agar plates and samples cut 6 mm in diameter were placed on the agar surface. After incubation, the zone of inhibition was determined by measuring the inhibition zone diameters around the discs. All experiments were performed in triplicate, and the results were expressed as mean and ± standard deviation.

### 2.13 MTT Test

The cytotoxicity of MNs was evaluated using the extract method according to the International Standard ISO 10993–5. The materials were prepared according to ISO 10993–12 standards and sterilized for 60 min on both sides with UV light. The sterilized MNs were added to the complete culture medium (Dulbecco’s Modified Eagle’s Medium-Low (DMEM-Low (Capricorn)) with 10% fetal calf serum (FBS, Biowest) and 1% penicillin/streptomycin (Capricorn)). MNs were incubated with the culture medium for 72 h at 37 °C and sample extracts were obtained. Meanwhile, the mouse fibroblast cell line (L929) (ATCC, CCL-1) was seeded in 96-well plates at 5 × 10^3^ per well and kept in a 5% CO_2_ incubator for 24 h. After incubation, the culture medium was removed from the cells, and previously prepared sample extracts were added to healthy cell lines. Negative and positive controls were defined by replacing the medium with fresh culture medium or fresh culture medium with 10% dimethyl sulfoxide (DMSO). After 1, 4, and 7 days of extract incubation, cell viability was assessed using the MTT (3-(4,5-Dimethylthiazol-2-yl)-2,5-Diphenyltetrazolium Bromide) kit (ElabScience). MTT solution was added and incubated for 3 h. DMSO was then added to precipitate formazan crystals, and the results were measured at 570 nm with a microplate reader (Agilent BioTek, Epoch). The absorbance values obtained were directly proportional to the number of viable cells and the percentage of viable cells was calculated by comparing with the negative control using the following eq 4

4 $$\%\mathrm{Viability}=\frac{{\mathrm{OD}}_{\mathrm{sample}}}{{\mathrm{OD}}_{\mathrm{control}}}\times 100$$

where OD_sample_ represents the mean optical density for the materials while OD_control_ represents the mean optical density for the negative control.^32^

### 2.14 Modeling of the Diabetic Environment

The effects of the microneedle samples on modeled diabetic environment cell viability were evaluated using an indirect MTT assay. For this purpose, extracts of the sterilized microneedle samples were prepared in accordance with the ISO 10993–12 standard.^33^ All experiments were conducted under high-glucose (35 mM) conditions to mimic the microenvironment of diabetic foot ulcers (DFU).^34^

Briefly, microneedle samples were incubated in serum-free Dulbecco’s Modified Eagle Medium High Glucose (DMEM-HG) at a concentration of 0.2 g/mL at 37 °C for 72 h. At the end of the incubation period, the conditioned media were collected, filtered through a 0.22 μm membrane filter, and supplemented with 10% fetal bovine serum (FBS) prior to use in cell culture experiments.

Human immortalized keratinocyte cells (HaCaT) were cultured in DMEM/F-12 medium supplemented with 10% FBS, 1% l-glutamine, and 35 mM glucose at 37 °C in a humidified atmosphere containing 5% CO_2_. For the viability assay, cells were seeded into 96-well plates at a density of 1 × 10^4^ cells per well and incubated overnight to allow cell attachment. Following incubation, the culture medium was removed and replaced with biomaterial extracts prepared under high-glucose conditions.

Cells cultured with complete medium alone served as the negative control, while dimethyl sulfoxide (DMSO) was used as the positive control. After 24 h of incubation, MTT solution was added to each well to achieve a final concentration of 0.5 mg/mL, and the plates were incubated at 37 °C for an additional 4 h. The formazan crystals formed by metabolically active cells were dissolved in DMSO following removal of the supernatant.

Optical density (OD) values were measured at 570 nm using a microplate reader. Cell viability was calculated as a percentage relative to the control group and expressed as mean ± standard deviation (SD). All experiments were performed in at least three independent replicates.

### 2.15 *In Vitro* Oxidative Stress Analysis of Samples Using HaCaT Cells

HaCaT cells were cultured in DMEM High Glucose (DMEM-HG) medium supplemented with 10% fetal bovine serum (FBS), 1% l-glutamine, 1% penicillin/streptomycin, and 1% nonessential amino acids at 37 °C in a humidified atmosphere containing 5% CO_2_. Cells were seeded into 24-well plates at a density of 1 × 10^5^ cells/mL and incubated for 24 h.

Following incubation, the microneedle samples were placed directly into wells containing HaCaT cells, and the cells were incubated with the samples for 72 h. At the end of the incubation period, the cells and samples were incubated at −80 °C for 1 h to facilitate the release of cellular contents. Subsequently, the culture media were collected and centrifuged at 5000 rpm for 15 min to obtain the supernatants.

The antioxidant and oxidant levels in the supernatants obtained from stressed HaCaT cells were determined using the Elabscience Total Antioxidant Status (TAS) Colorimetric Assay Kit (E-BC-K801-M) and the Elabscience Total Oxidant Status (TOS) Colorimetric Assay Kit (E-BC-K802-M), respectively, in accordance with the manufacturer’s instructions.

Oxidative stress levels were evaluated by calculating the oxidative stress index (OSI), as described in the literature. OSI was calculated as the ratio of TOS to TAS using the following formula

5 $$\mathrm{OSI}=\frac{\mathrm{TOS}\;\left(\mathrm{\mu mol}\;H_{2}O_{2}\;\mathrm{equiv}/L\right)}{\mathrm{TAS}\;\left(\mathrm{mmol}\;\mathrm{Trolox}\;\mathrm{equiv}/L\right)\times 10}$$

An increase in OSI values was interpreted as an indication of elevated oxidative stress.

### 2.16 Scratch Assay Analysis Under Stimulated Diabetic Foot Ulcer Microenvironment

The migratory capacity of HaCaT human keratinocytes under simulated hyperglycemic conditions was assessed using a scratch wound gap closure assay. HaCaT cells were seeded at a density of 3 × 10^5^ cells per well in 6-well tissue culture plates and cultured in high-glucose Dulbecco’s Modified Eagle’s Medium (DMEM-HG, 35 mM d-glucose) supplemented with 10% fetal bovine serum (FBS) and 1% penicillin/streptomycin at 37 °C and 5% CO_2_ until a confluent monolayer was formed (approximately 24 h). A uniform scratch wound was introduced across the center of each confluent monolayer using an AutoScratch device (Agilent BioTek, Winooski, VT, USA). Detached cells and debris were removed by washing twice with phosphate-buffered saline (PBS, pH 7.4), and fresh DMEM-HG containing 10% FBS was added.

Microneedle sample extracts were prepared in the same way as the Keratinocyte MTT analyses (incubated in serum-free Dulbecco’s Modified Eagle Medium High Glucose (DMEM-HG) at a concentration of 0.2 g/mL at 37 °C for 72 h).^47^ Following scratch wound creation, cells were treated with the respective extracts for 24 h. An untreated HaCaT monolayer maintained under identical hyperglycemic culture conditions served as the negative control. Microscopic images of the scratch wound area were acquired immediately after scratch creation (0 h) and at 24 h using an inverted phase-contrast microscope at 5× magnification. All experiments were performed in triplicate (*n* = 3).

Wound closure area at each time point was quantified using ImageJ software (National Institutes of Health, USA) with the MRI Wound Healing Tool plugin. The percentage wound closure was calculated according to the following equation

6 $$Wound\,\;closure\;\left(\%\right)=\frac{wound\;area\;at\;0\;h-wound\;area\;at\;\mathrm{24}\;h}{wound\;area\;at\;0\;h}\times 100$$

### 2.17 Penetration Testing

For this study, the penetration abilities of MNs were evaluated using parafilm, due to parafilm’s similarity to pig skin.^35^ The parafilm, with each layer measuring approximately 127 μm in thickness, was folded into 8 layers to form a stack of about 1 mm. The MNs were then inserted into the Parafilm stack by manual thumb pressure and subsequently removed. The layers were carefully separated one by one, and the number of holes and the hole width in each layer were evaluated using an optical microscope (Olympus AnalySIS, USA).

### 2.18 Statistical Analysis

Experiments were performed in triplicate, and data was expressed as mean ± standard deviation (SD). Statistical analyses were conducted using one-way ANOVA followed by Tukey’s post hoc test to compare differences among groups. Appropriate symbols were used to indicate the *p*-values in the graphs. A *p*-value <0.05 was considered statistically significant, while *p* < 0.01 and *p* < 0.001 were reported as highly significant and very highly significant, respectively.

## 3 Results and Discussion

### 3.1 Characterization and Substitution Degree of GelMA

The polymer modification process of gelatin to GelMA adds methacryloyl groups to the gelatin polymer. To determine the successful addition of these groups and to calculate the degree of substitution, ^1^H NMR and FT-IR analysis was conducted on both gelatin and GelMA given in Figure 1.

**Figure 1:**
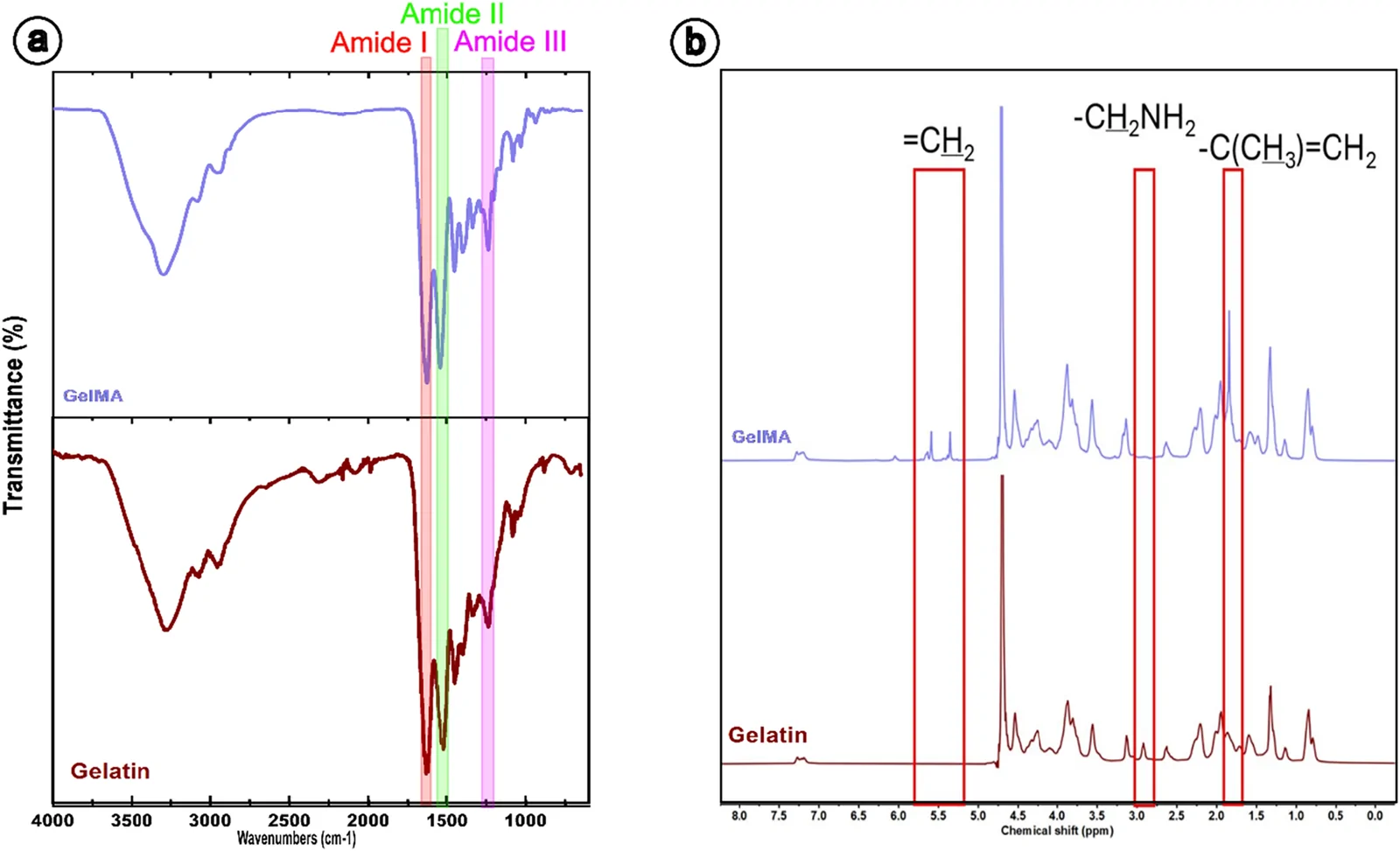
FT-IR (a) and ^1^H NMR (b) spectra of GelMA and gelatin.

In the FT-IR spectra, both gelatin and GelMA exhibited characteristic amide bands, including Amide I (∼1650 cm^–1^, C=O stretching), Amide II (∼1550 cm^–1^, N–H bending), and Amide III (∼1240 cm^–1^, C–N stretching). These peaks are consistent with the peptide backbone of gelatin. GelMA spectra, in comparison to gelatin spectra, shows shifts and changes, indicating methacrylation of gelatin was successful.^36^ For the ^1^H NMR spectra new peaks were observed between 5.2 and 5.8 ppm. This is due to the C=C bonds coming from the vinyl groups of the anhydride in the added methacrylic anhydride. The major increase in the peak around 1.8 ppm can be attributed to the methyl protons of the added methacryloyl groups. In contrast the characteristic lysine methylene resonance around 2.9 ppm, present in gelatin, was markedly reduced in GelMA, confirming that lysine residues were substituted by methacryloyl groups. The degree of substitution (DS) was calculated using the lysine reference peak according to eq ([Disp-formula eq1]) yielding a DS of 92.1%. This high substitution degree falls within the range reported for GelMA depending on reaction conditions.^25,37^ Higher GelMA substitution degrees correlate with enhanced mechanical strength, improved tip integrity during skin insertion, and greater structural stability under compression.^38^

### 3.2 FT-IR Characterization of Drug-Loaded Microneedles and Particles

The FT-IR spectra of PLA, metformin, amoxicillin, salicylic acid, GelMA MN, amoxicillin loaded GelMA, metformin loaded GelMA, and sprayed PLA/SA particles were given in Figure 2.

**Figure 2:**
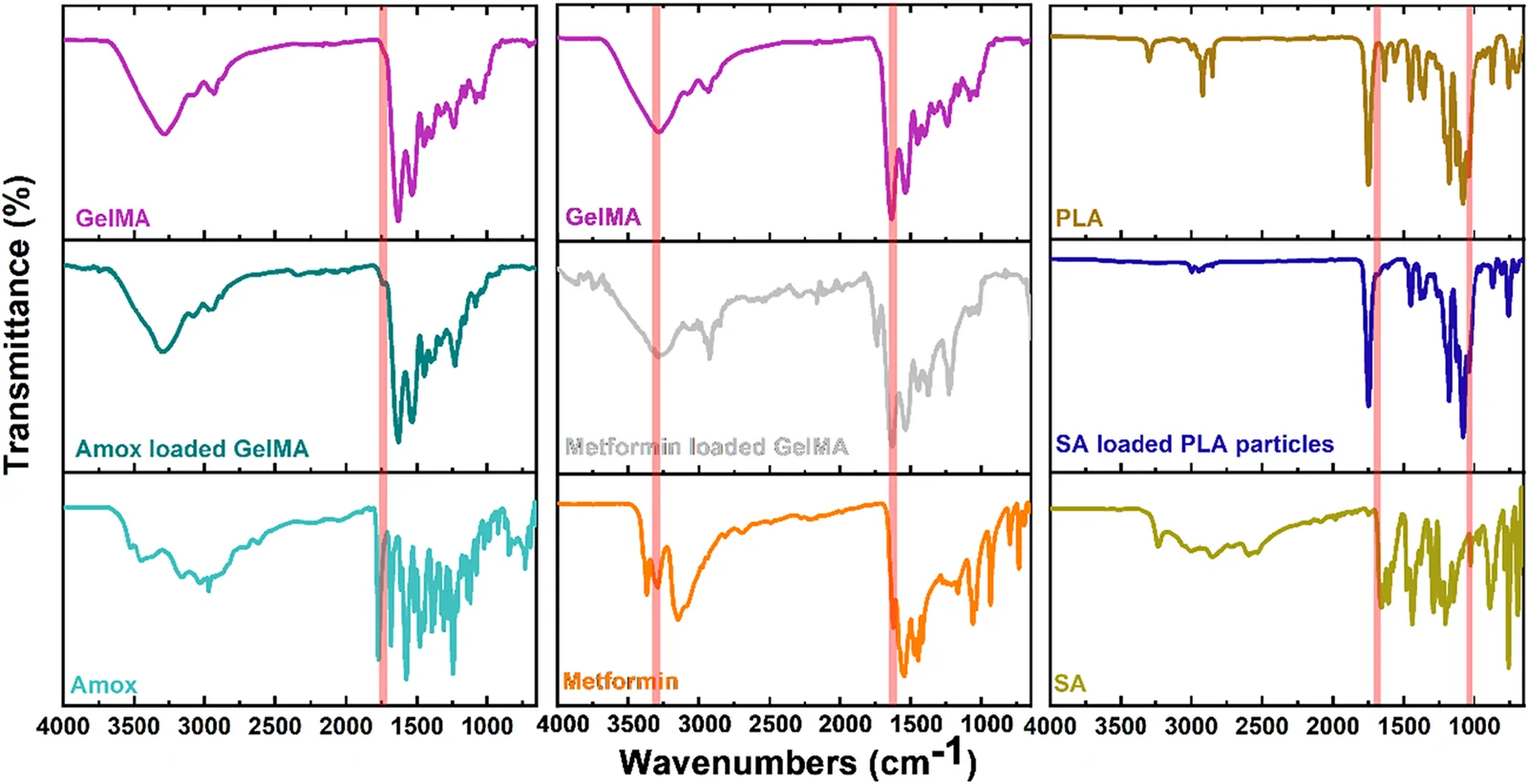
FT-IR spectra of amox, metformin, salicylic acid (SA), GelMA, PLA, amox loaded GelMA, metformin loaded GelMA and SA loaded PLA particles.

Amoxicillin gives loads of peaks between the ranges of 1800 and 1200 cm^–1^. Major peaks in these areas are ∼1770 cm^–1^ for C=O stretching in −COOH group, ∼1680 cm^–1^ for C=O amide stretching, 1580 cm^–1^ for C–C ring and 1390 cm^–1^ for C–N stretching.^39^ GelMA showed typical absorption bands at ∼3200–3400 cm^–1^ (−OH and N–H stretching), ∼1630 cm^–1^ (Amide I, C=O stretching), ∼1550 cm^–1^ (Amide II, N–H bending), and ∼1250 cm^–1^ (Amide III, C–N stretching).^40^

Metformin has peaks in 3350 and 3250 cm^–1^, these peaks can be attributed to symmetric and asymmetric stretching’s of N–H bonds respectively. Peak in ∼3150 cm^–1^ is for asymmetric stretching of C–H bonds. ∼1620 cm^–1^ peak gives C=N stretching vibrations and peaks between ∼1000 and 1100 cm^–1^ gives C–N stretching vibrations, in agreement with reported data.^41^ In SA ∼3250 cm^–1^ and peak can be observed, this peak gives ∼O–H cm^–1^ stretching, ∼2860 cm^–1^ gives C–H stretching, ∼1600 cm^–1^ gives C=O stretching and ∼1440 cm^–1^ gives C–C stretching respectively.^42^ PLA spectrum gives peaks at ∼2900 cm^–1^ for C–H stretching, ∼1750 cm^–1^ for C=O stretching, ∼1450 and ∼1350 cm^–1^ for CH_3_ symmetric bending, ∼1080 cm^–1^ for C–O–C stretching, consistent with literature.^43^

In the drug-loaded systems, characteristic peaks of the drugs were observed alongside the backbone spectra of GelMA or PLA. For instance, amoxicillin-loaded GelMA retained the GelMA amide peaks with additional signals in the 1770–1580 cm^–1^ region from amoxicillin. Similarly, metformin-loaded GelMA displayed both the GelMA amide bands and metformin-associated peaks (∼1620 cm^–1^ for C=N and 3350–3250 cm^–1^ for N–H stretching). The FT-IR spectrum of SA-loaded PLA particles exhibited the PLA backbone peaks (1750 cm^–1^ C=O and 1080 cm^–1^ C–O–C) together with the O–H and C=O peaks of SA. These findings support the successful incorporation of the drugs into the corresponding GelMA or PLA-based matrices.

### 3.3 SEM Analysis

The morphology of the produced GelMA MNs was examined by SEM, as shown in Figure 3. GelMA-MN (a), A-M-GelMA-MN (b), A-M-GelMA-MN@SA (c) provided smooth and uniform microneedle images. Due to the process of producing our MNs includes stopping the DLP printer halfway and restarting with a new solution, proper and homogeneous observation of the attachment region of the MNs to the base layer is crucial.^44^ This layer can be observed as perfect in the visuals of Figure 3a–c as there are no gaps between the MNs and the base layer.

**Figure 3:**
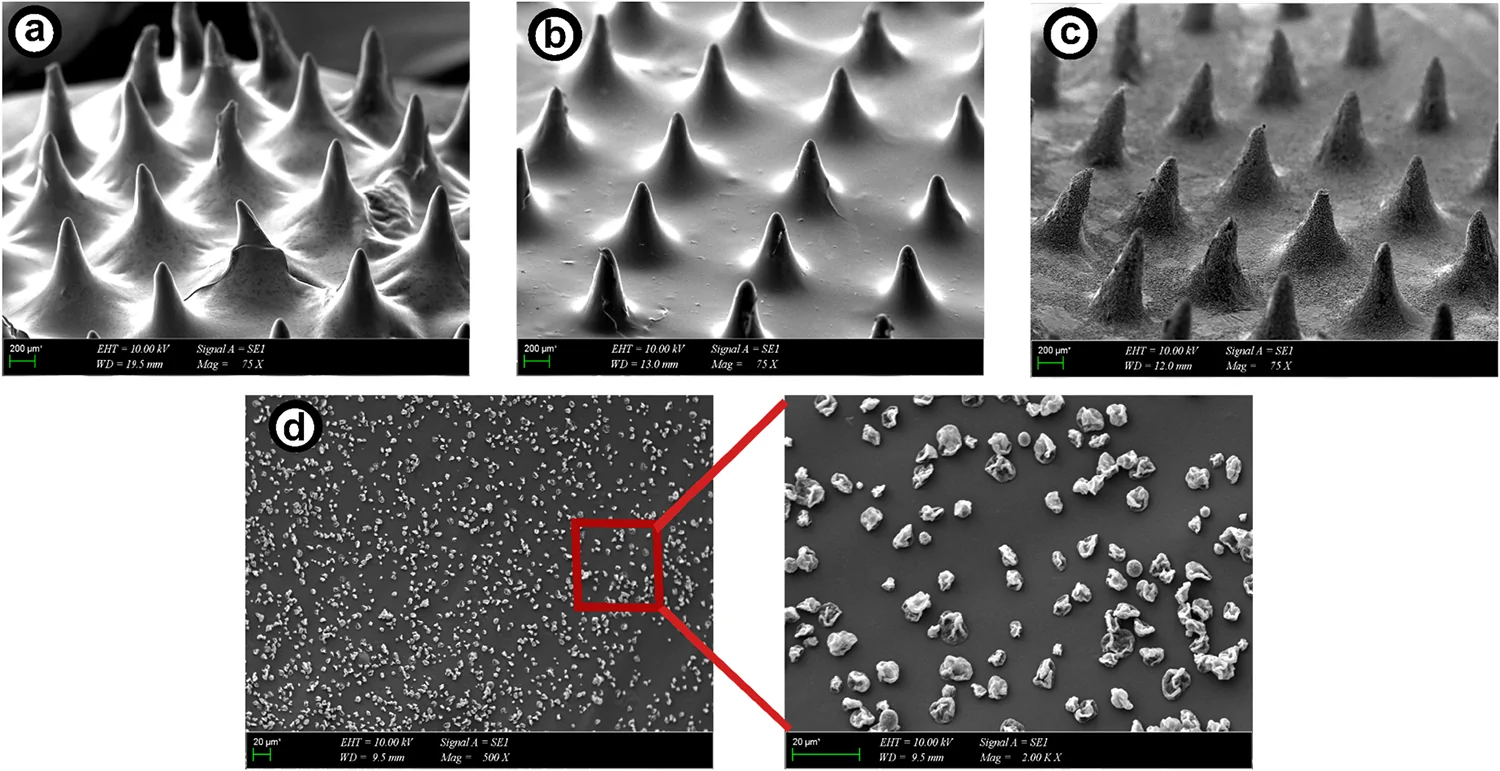
Scanning electron micrographs of the produced MNs (75×), GelMA-MN (a), A-M-GelMA-MN (b), A-M-GelMA-MN@SA (c), sprayed PLA/SA microparticles (500×) and their enhanced visual (2000×) (d).

For MN production it is crucial to know the height of the needle size.^27^ Based on the height of the microneedle we can estimate the thickness of skin that the microneedle will penetrate. With SEM visualization the determined height of the MNs was measured to be 615.55 ± 0.636 μm for GelMA-MN, 624.37 ± 5.553 μm for A-M-GelMA-MN and 626.725 ± 2.252 μm for A-M-GelMA-MN@SA. These values are consistent with previous reports of GelMA-based MNs designed for efficient skin penetration.^27^ The discrepancy between the nominal CAD microneedle height and the measured fabricated height effect is consistent with our group’s prior optimization work using the same printing approach, where increasing the design height (from 500 to 1000 μm) was accompanied by a progressively larger absolute shortfall between design and measured height.^12^ Given the substantially taller design height used here (2000 μm) relative to our prior optimization range (500–1000 μm), a proportionally larger shortfall is consistent with this same mechanism.

In addition, electrosprayed PLA/SA microparticles were successfully produced and visualized under 500× and 2000× magnifications (Figure 3d). The average particle diameter was calculated using ImageJ program and were determined as 1.504 ± 0.369 μm. This particle size is in agreement with values reported for PLA microparticles prepared under similar conditions.^19^ The produced micronized particles of PLA/SA can be easily transported between the skin cells, leading to better utilization of SA’s antioxidant properties.^45^

### 3.4 DSC Analysis

GelMA-MN (a), A-M-GelMA-MN (b), A-M-GelMA-MN@SA (c) samples were thermally characterized given in Figure 4. The physicochemical properties of GelMA depend on both the source of gelatin and the level of methacrylation.^46^ It has been reported that GelMA has an overlapping Tm value around ∼220.00 °C, attributed to its peptide backbone.^47^ Pure metformin shows a sharp melting endotherm at 231 °C^48^ Amoxicillin at 130 °C^49^ and PLA at 166.7 °C.^50^

**Figure 4:**
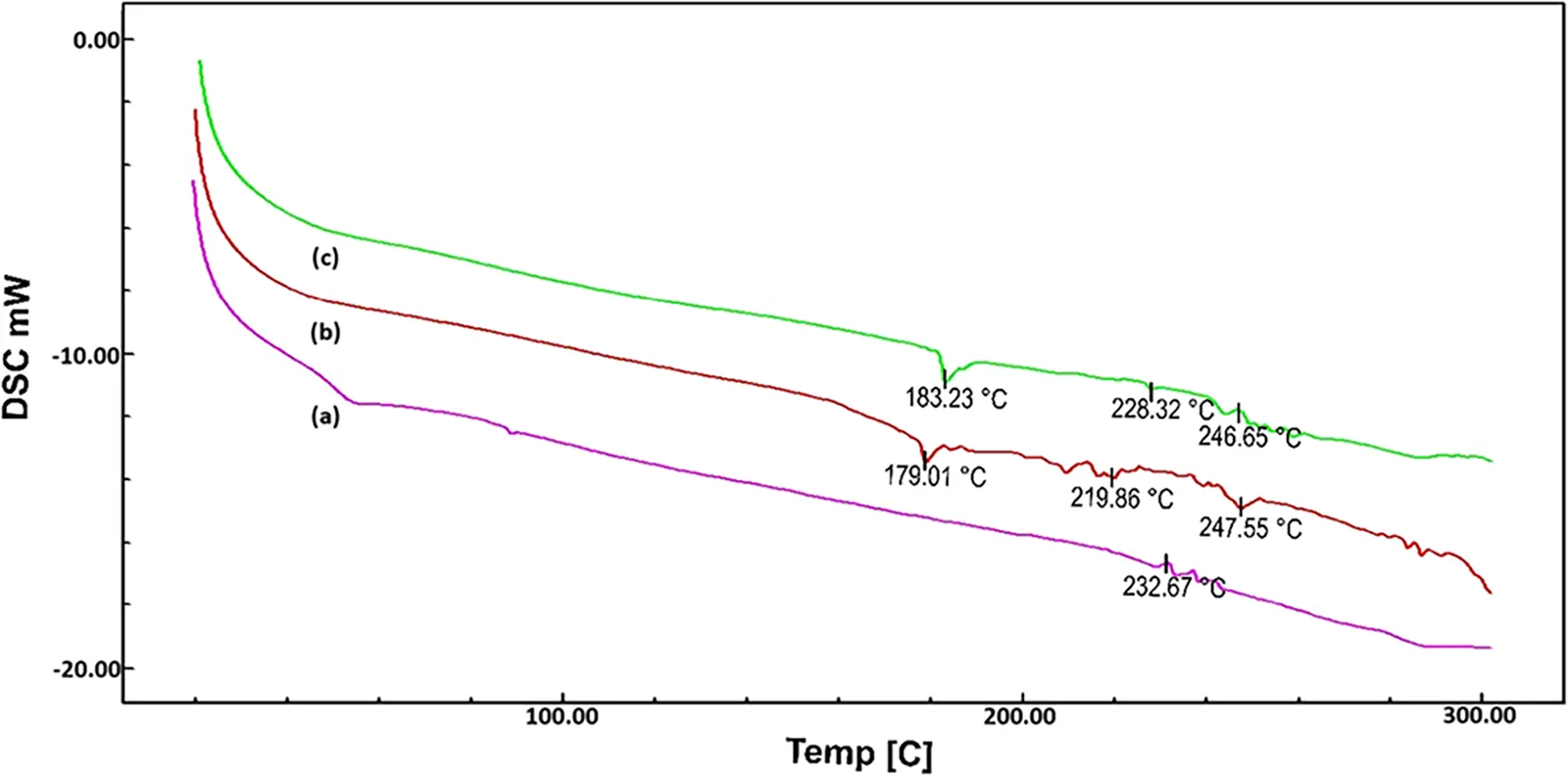
DSC curves of the MNs. GelMA-MN (a), A-M-GelMA-MN (b), A-M-GelMA-MN@SA (c).

In our study, GelMA-MN displayed two transition peaks: an endothermic event at 52.44 °C, corresponding to moisture loss, and a major peak at 232.67 °C, associated with collagen melting. For the A-M-GelMA-MN material, three peaks were observed at 179.01 °C, 219.86 °C, and 247.55 °C (Figure 4). These are assumed to be the characteristic Tm peaks of Metformin and Amoxicillin present in the GelMA matrix. Similarly, for the A-M-GelMA-MN@SA composite exhibited peaks at 183.23 °C, 228.32 °C, and 246.65 °C. The observed shifts and broadening of thermal events suggest altered thermal behavior and possible matrix–drug interactions after drug loading. Although peak overlap and drug dilution may also explain these observations. However, these DSC results should be interpreted as supportive evidence rather than definitive proof of improved thermal stability. Overall, DSC analysis supported the presence of the incorporated components within the GelMA/PLA-based microneedle system and indicated possible interactions between the drugs and polymeric matrices. However, DSC alone cannot establish the specific molecular basis of these interactions; techniques such as FTIR, XRD, or NMR would be required to confirm hydrogen bonding or ionic mechanisms directly

### 3.5 Mechanical Strength

Mechanical analysis was conducted on GelMA MN, Amox loaded GelMA and Metformin loaded GelMA cylindrical samples. Since sufficient mechanical strength is essential for MNs to penetrate the skin effectively, the mechanical performance of GelMA-MN was compared with amoxicillin and metformin loaded MNs. The mechanical strength of GelMA-MN was measured to be approximately 1.3 MPa, while Amoxicillin-loaded GelMA MNs exhibited a slightly lower strength of approximately 1.2 MPa (Figure 5a). This indicates that amoxicillin may slightly weaken the mechanical strength due to its interaction with the polymer matrix. On the other hand, GelMA MNs with Metformin exhibited a strength of approximately 1.3 MPa, similar to GelMA-MN, indicating that metformin loading has no unfavorable effect on maintaining mechanical integrity (Figure 5a).

**Figure 5:**
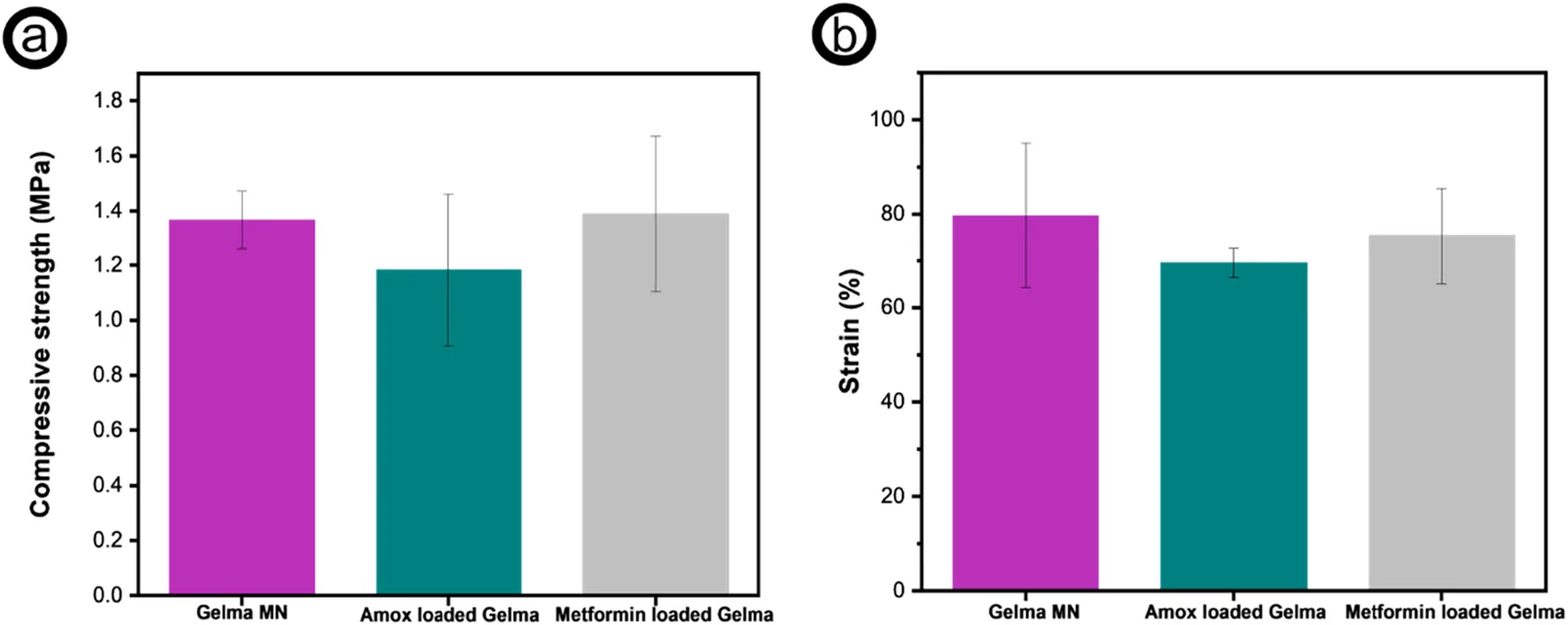
Compressive strength (a) and strain of the layers (b).

Analysis of the deformation (strain) data shown in Figure 5b revealed that GelMA-MN exhibited a maximum strain of approximately 80%, indicating the highest elasticity. The addition of amoxicillin caused this value to drop to ∼70%, while metformin-loaded MNs maintained similar elasticity with ∼75% strain Figure 5b. This may be attributed to metformin’s homogeneous distribution within the GelMA matrix, which preserves the matrix intrinsic elastic properties compared to the amoxicillin-loaded platform. In conclusion, all cylindrical specimens of the groups exhibited adequate mechanical performance for microneedle applications.^51^ However, Amoxicillin loading resulted in a slight reduction in mechanical strength, whereas Metformin loading did not adversely affect the structural integrity or elasticity. These findings indicate that Metformin-loaded GelMA MNs maintain mechanical robustness and are well-suited for transdermal applications.

### 3.6 Swelling and Degradation Behaviors of the Microneedle

The biodegradability of MN systems was evaluated under *in vitro* conditions over a period of 30 days. The degradation profiles for GelMA-MN, A-M-GelMA-MN, and A-M-GelMA-MN@SA are given in Figure 6d. Degradation percentages increased over time in all groups. On days 1 and 5 (for day 5, 28.26% ± 1.17, 26.31% ± 2.11, 27.15% ± 0.91, respectively), degradation rates showed no significant differences, indicating comparable initial stability across all microneedle systems.

**Figure 6:**
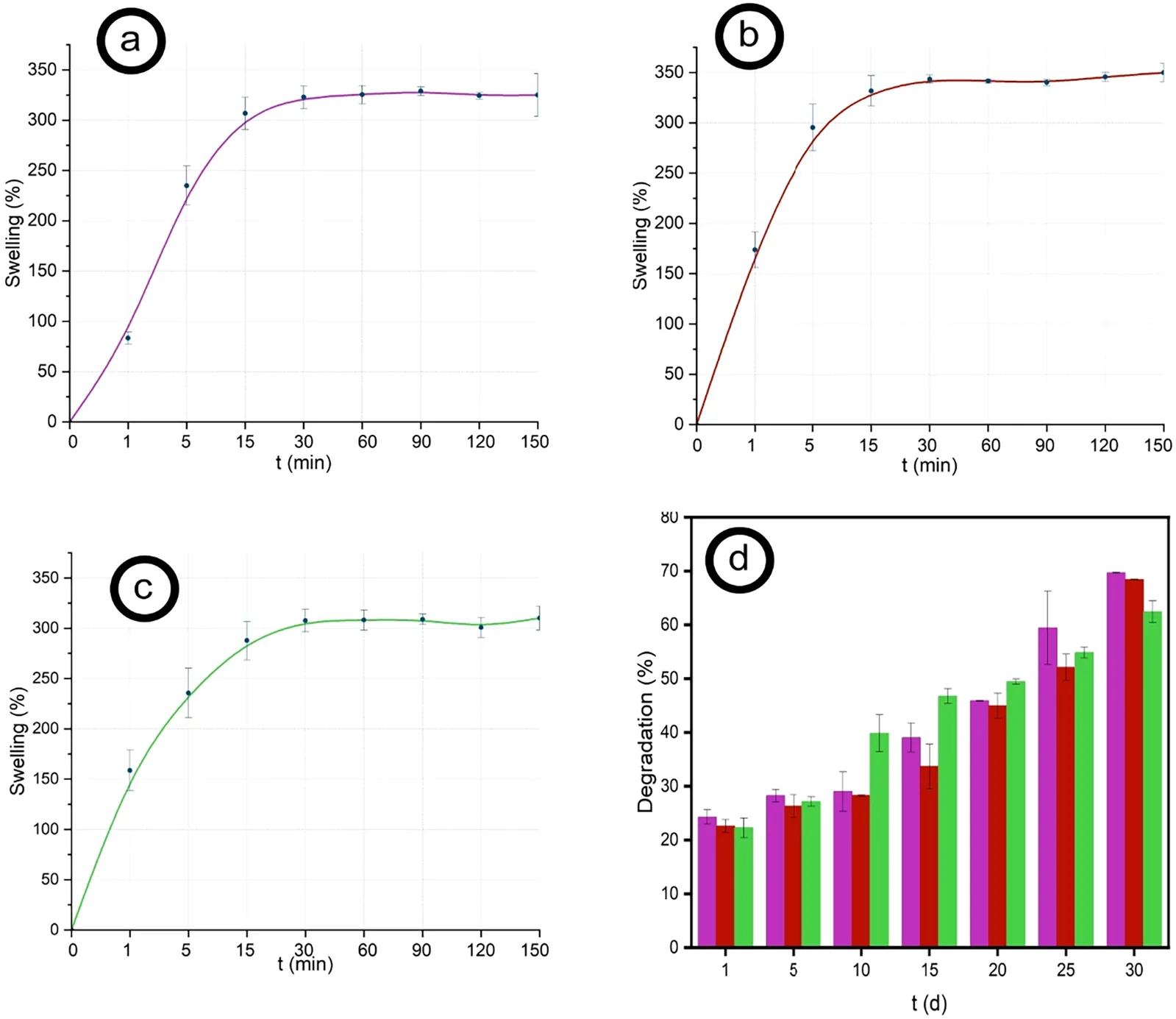
Swelling graphs of the MNs. GelMA-MN (a), A-M-GelMA-MN (b), A-M-GelMA-MN@SA (c). Degradation graphs of the samples GelMA-MN (purple), A-M-GelMA-MN (red), A-M-GelMA-MN@SA (green) (d).

After day 5, a marked increase in degradation was observed in all groups. By day 30, degradation rates reached approximately 69.67% ± 0.16 for GelMA-MN, 68.42% ± 0.12 for A-M-GelMA-MN, and 62.41% ± 2.04 for A-M-GelMA-MN@SA. The A-M-GelMA-MN@SA group exhibited a slightly lower overall degradation than the other formulations. Although hydrolytic degradation of PLA can locally lower pH and accelerate acid-catalyzed hydrolysis of the sample.^52^ the PLA microparticles may simultaneously act as physical fillers that stabilize the surrounding GelMA network and slow bulk erosion. Intermolecular interactions between SA and the GelMA matrix may also contribute to a more compact structure, reducing the rate of mass loss.

Despite these differences, which were most apparent within the first 15 days, all groups reached comparable degradation levels by day 30. Drug loading had only a minimal effect on the overall degradation profile, and the biodegradable character of all MN systems was confirmed.^53^

The swelling behavior of the MNs in an aqueous environment was evaluated to compare the hydrophilicity and matrix structural properties of the formulations. To evaluate the hydrophilicity and matrix structure of different formulations, the swelling behavior of MNs in an aqueous environment was examined. All groups exhibited a rapid initial swelling response, with the most pronounced increase occurring within the first 15 min with mean values of 332.07 ± 21.26% (GelMA-MN), 355.01 ± 9.43% (A-M-GelMA-MN), and 323.15 ± 11.87% (A-M-GelMA-MN@SA), reflecting the high-water absorption capacity of the hydrogel matrix (Figure 6a–c). Among these, the A-M-GelMA-MN@SA group (Figure 6c) exhibited the highest swelling rate in as little as 5 min, reached the maximum swelling percentage in approximately 15 min, and maintained this high level for up to 60 min. This may indicate that the inclusion of PLA and SA particles creates a looser, more porous network, thereby facilitating water diffusion.

In contrast, the GelMA-MN (Figure 6a) exhibited a slower and lower swelling profile, consistent with a denser cross-linked network and lower water absorption capacity. The A-M-GelMA-MN (Figure 6b) exhibited a moderate profile characterized by a rapid initial swelling phase followed by a slower increase rate; this may indicate that drug-matrix interactions enhance water retention capacity but limit maximum swelling capacity due to the more compact structure. In conclusion, while all groups eventually reached a plateau in their swelling behavior, the drug-loaded formulations, particularly A-M-GelMA-MN@SA group, exhibited higher and faster swelling behavior. These findings indicate a structural feature that may provide significant advantages in terms of drug release and skin penetration.^12^

### 3.7 Drug Release Behavior of the Microneedle

Since the UV absorbance spectra of the three drugs showed overlap, the release profiles were obtained from layer-specific single-drug-loaded model formulations rather than from simultaneous quantification of the complete multilayer construct. Maximum absorbance values were observed at 228, 232, and 298 nm for Amoxicillin, Metformin, and Salicylic acid, respectively (Figure S1). The Amoxicillin release profile (Figure 7a) exhibited a typical biphasic pattern. An initial burst release occurred during the first 6 h, with approximately 45% of the drug rapidly released, likely due to molecules loosely bound or located near the microneedle surface. This was followed by a slower, sustained release phase, reaching ∼78% at 24 h and approaching complete release (>99%) by the end of the 72 h study period. Similarly, it was observed that ∼42% of Metformin was released within the first 6 h, exceeding 80% release within 24 h, and was completely achieved within 72 h (Figure 7b). In contrast, it is noteworthy that SA was dramatically released (∼60%) from the MN platform within 3.5 h (Figure 7c).

**Figure 7:**
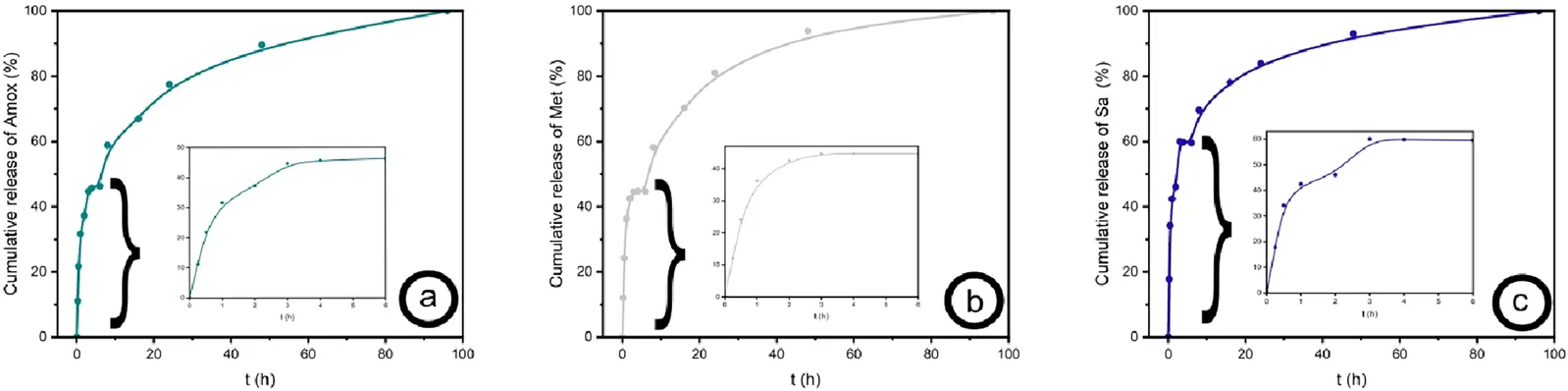
Release profiles of the drug loaded layers, amoxicillin loaded GelMA (a), metformin loaded (b) GelMA, SA loaded PLA particles (c).

The biphasic release profiles of amoxicillin and metformin initially exhibit rapid drug release followed by a prolonged release phase. This is a characteristic feature of controlled drug delivery systems designed to achieve both immediate and long-term therapeutic effects. The initial burst release can be attributed to the diffusion of drug molecules located on or near the microneedle surface, which provides an early onset of efficacy.^54^ Subsequently, the slower release phase is likely driven by the gradual diffusion of the drug from the microneedle matrix, ensuring the drug’s prolonged availability.^55^ The burst release of SA can be explained by differences in the physicochemical properties of the substance, such as solubility and molecular weight, as well as possible differences in polymer interactions and the porosity of the microneedle matrix.^56^ Overall, the release behavior of the platform indicates the versatility of the microneedle platform in delivering drugs with diverse release kinetics.

### 3.8 Antibacterial Testing

The antibacterial properties of GelMA-MN, A-M-GelMA-MN, A-M-GelMA-MN@SA were examined using the disk diffusion method. The results showed that GelMA-MN does not possess antibacterial properties against Gram-positive and Gram-negative bacteria (Figure 8). Loading with amoxicillin conferred antibacterial activity against both *E. coli* and *S. aureus*, with inhibition zones of 20 ± 0.5 mm and 21 ± 2.5 mm, respectively (Table 3). These values are comparable to those reported by Erkus et al. for amoxicillin-loaded GelMA MNs (22 mm against *S. aureus* and 25 mm against *E. coli*).^27^ Coating these MNs with SA-loaded PLA microparticles resulted in a moderate increase in inhibition zone diameter (22 ± 1.5 mm against *E. coli* and 23 ± 0.5 mm against *S. aureus*), consistent with the known antibacterial properties of SA.^57^ Although the absolute increase in zone diameter was modest (∼2 mm for both strains), the trend supports an additive antibacterial contribution from the SA-containing surface layer. The unloaded GelMA-MN group showed no detectable antibacterial activity against either strain, confirming that the observed effects were attributable to the loaded agents.

**Figure 8:**
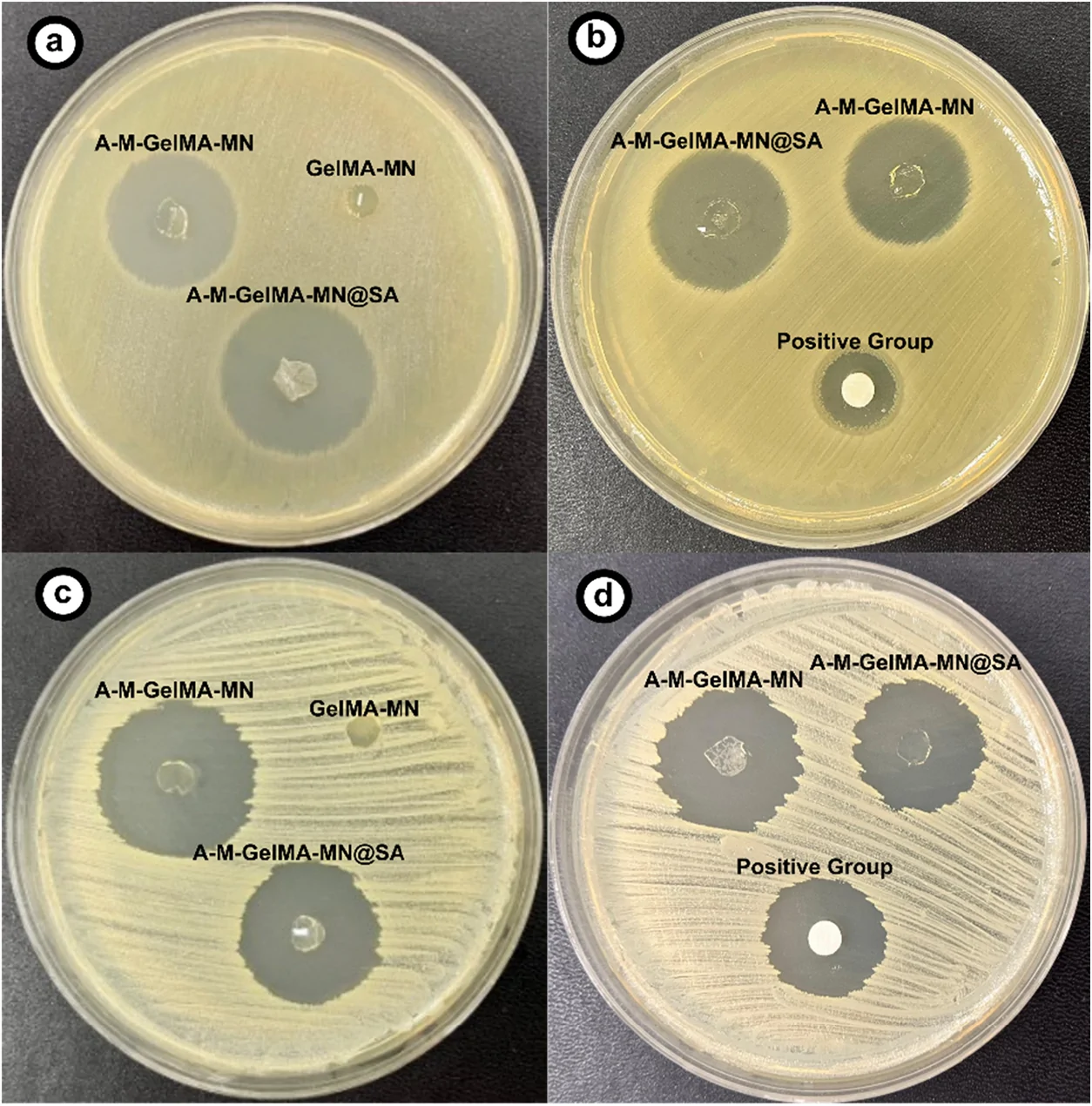
Inhibition zones of MNs. GelMA-MN, A-M-GelMA-MN, A-M-GelMA-MN@SA. Positive group (Amoxicillin/25 μg), for *E. coli* (a, b) and *S. aureus* (c, d) respectively.

**Table 3:** Inhibition Zone Diameters (mm) of the Produced MN Samples

	GelMA-MN	A-M-GelMA-MN	A-M-GelMA-MN@SA	Positive control
*E. coli*	-	20 ± 0.5	22 ± 1.5	12
*S. aureus*	-	21 ± 2.5	23 ± 0.5	19

### 3.9 MTT Assay

The biocompatibility properties of MN platforms were investigated in the L929 cell line (Figure 9). Cell viability was found to decrease slightly from day 1 to 4 in the GelMA-MN, A-M-GelMA-MN, A-M-GelMA-MN@SA groups. However, over the following 3 days, the decrease in viability was more pronounced in all groups, although it remained at a favorable level in terms of biocompatibility. The GelMA-MN group had the highest cell viability, while viability decreased in the A-M-GelMA-MN group and declined more in the A-M-GelMA-MN@SA group. Although a decrease in cell viability was observed compared to the negative control group, the viabilities were 95%, 85%, and 82%, respectively, and were at nontoxic levels.^32^ The inclusion of SA in GelMA MNs led to a decrease in cell viability. This is coherent with previous reports indicating that SA can exhibit antiproliferative effects in certain mammalian cell lines, depending on its concentration and exposure time.^58^ Collectively, these results confirmed that GelMA MNs possess high biocompatibility and can deliver multiple bioactive agents with minimal impact on cell viability.

**Figure 9:**
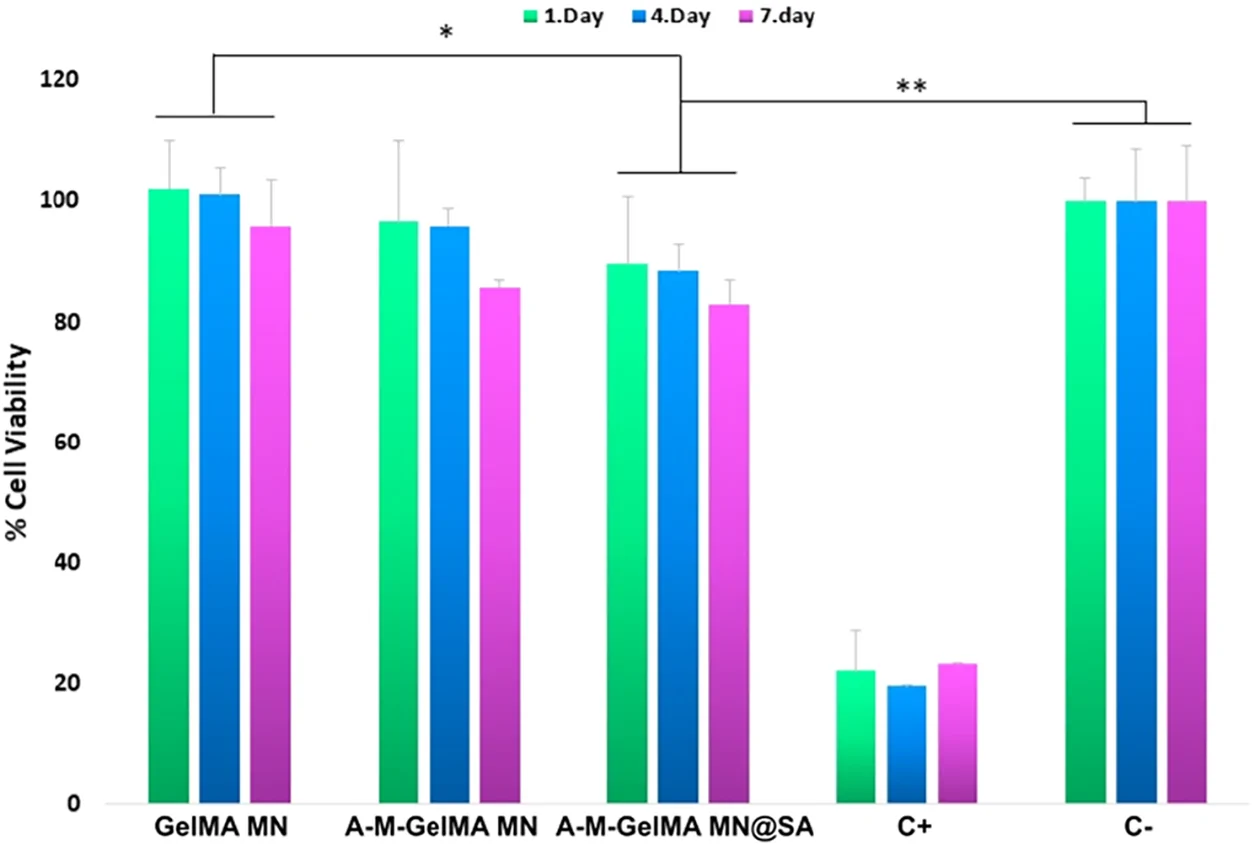
Percentage of indirect cell viability results for three different MN. Statistical significance “*p*” is shown as *p* < 0.05 = “*” and *p* < 0.01 = “**”. Negative control: complete culture medium alone; positive control: culture medium with 10% DMSO.

### 3.10 Diabetic Modeling

The biocompatibility of the MN formulations was evaluated using an MTT assay performed on HaCaT cell line, and the obtained results are presented in Figure 10. The GelMA MN formulation exhibited a cell viability of 93.08%, demonstrating the high biocompatibility of the GelMA-based matrix. Cell viability values of 81.68% and 77.98% were determined for the A-M-GelMA MN and A-M-GelMA MN@SA groups, respectively. Although a moderate reduction in cell viability was observed in the drug-loaded microneedle formulations compared to the GelMA MN group, all experimental groups-maintained cell viability levels above the 70% cytotoxicity threshold defined by ISO 10993–5. The moderate reduction is mostly due to the amoxicillin’s ROS-fibroblast effects, metformin’s AMPK/mTOR-mediated proliferation suppression and SA’s acidic/keratinolytic effects. These findings indicate that the developed microneedle systems did not induce significant cytotoxic effects on HaCaT cells.

**Figure 10:**
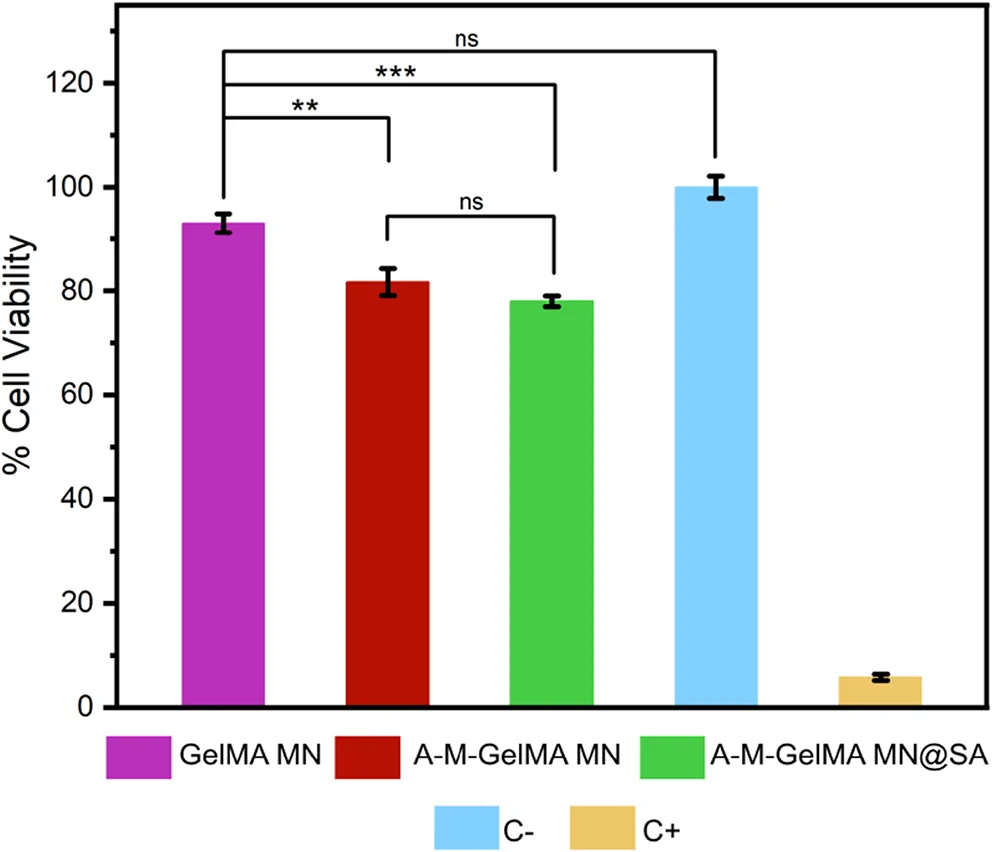
Cell viability of HaCaT keratinocytes cultured under hyperglycemic conditions (35 mM glucose) for 1 day following exposure to GelMA-MN, A-M-GelMA-MN, and A-M-GelMA-MN@SA, assessed by MTT assay and expressed as % cell viability relative to the negative control. C– (negative control): untreated cells in complete culture medium. C+ (positive control): cells treated with 10% DMSO. Data are presented as mean ± SD (*n* = 3). Statistical significance was determined by one-way ANOVA with Tukey’s post hoc test (Section 2.18); **p < 0.01, ***p < 0.001, ns = not significant.

The observed decrease in cellular metabolic activity in the drug-loaded groups is associated with the biological effects of the incorporated agents rather than direct induction of cell death. Amoxicillin, a β-lactam antibiotic, generally exhibits low toxicity toward mammalian cells; however, it has been reported that, under in vitro conditions and at high concentrations, amoxicillin may partially impair fibroblast migration by affecting reactive oxygen species (ROS) homeostasis.^59,60^ Metformin is known to suppress cell proliferation through activation of AMP-activated protein kinase (AMPK) and inhibition of the mTOR signaling pathway.^61^ Therefore, the reduction in cell viability detected in the MTT assay is attributed to regulation of metabolic activity and cell proliferation rate rather than cytotoxic cell death.^62,52^

The decrease in cell viability observed in the A-M-GelMA MN@SA group may be attributed to the acidic and keratinolytic properties of salicylic acid. Salicylic acid is an important therapeutic agent in diabetic foot ulcers, as it contributes to the softening of hyperkeratotic and callus tissue, thereby reducing mechanical pressure.^21,22^ However, due to its acidic nature, salicylic acid can influence cellular metabolism and, when applied at high or uncontrolled doses, may increase the risk of tissue damage, particularly in patients with peripheral neuropathy and impaired circulation. In the present study, the maintenance of cell viability above the cytotoxicity threshold suggests that salicylic acid was incorporated into the microneedle system at a biocompatible concentration.

Overall, these results demonstrate that the GelMA-based microneedle platform loaded with amoxicillin, metformin, and salicylic acid is capable of preserving keratinocyte viability while targeting key pathological components of diabetic foot ulcers, including infection, metabolic dysfunction, and mechanical barrier formation. These in vitro biocompatibility findings provide strong preliminary evidence supporting further in vivo evaluation of the developed system, particularly in terms of wound closure kinetics, histological tissue remodeling, and angiogenesis-related markers in diabetic wound models.

### 3.11 Antioxidant Assay

The oxidative stress analysis performed on HaCaT cells were given in Figure 11. The total oxidant status (TOS) value of the GelMA MN group was determined to be 80.4 ± 3.6 μmol. In the A-M-GelMA MN group, the TOS level decreased to 66.8 ± 3.1 μmol, while a further reduction to 54.6 ± 2.8 μmol was observed in the A-M-GelMA MN@SA group. In contrast, total antioxidant status (TAS) values were measured as 0.72 ± 0.03 mmol for the GelMA MN group, 0.82 ± 0.04 mmol for the A-M-GelMA MN group, and 0.91 ± 0.03 mmol for the A-M-GelMA MN@SA group. These data indicate that the drug-loaded microneedle systems reduced the oxidant burden while enhancing the cellular antioxidant capacity.

**Figure 11:**
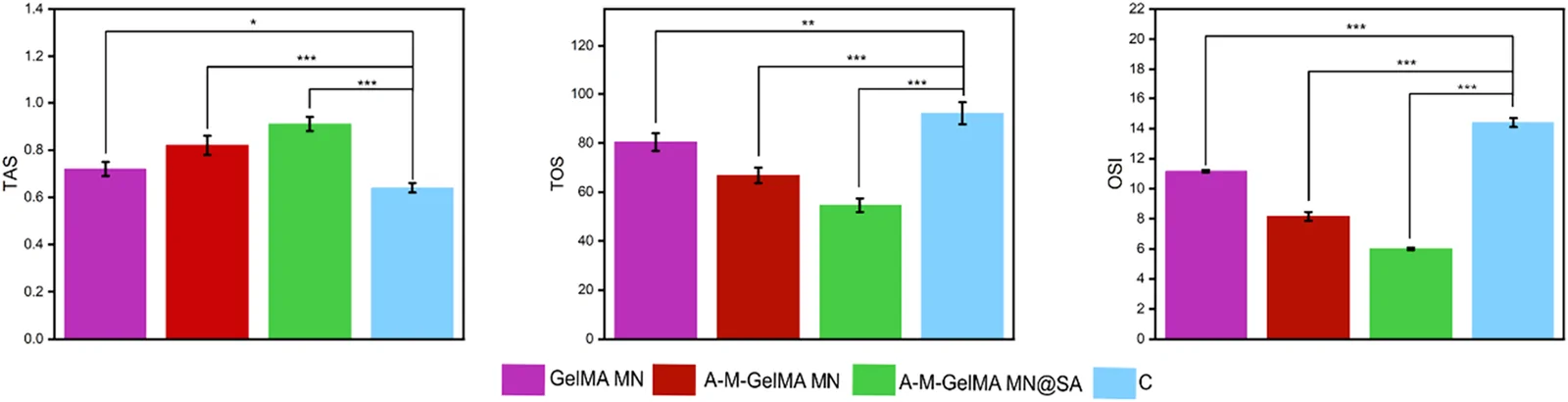
Oxidative stress modulation by the microneedle formulations, assessed on HaCaT cells under hyperglycemic conditions. Total antioxidant status (TAS, mmol), total oxidant status (TOS, μmol), oxidative stress index (OSI, calculated as TOS/[TAS × 10]). GelMA-MN, A-M-GelMA-MN, and A-M-GelMA-MN@SA are compared against untreated control (C). Data are presented as mean ± SD (*n* = 3). Statistical significance was determined by one-way ANOVA with Tukey’s post hoc test (Section 2.18); *p < 0.05, **p < 0.01, ***p < 0.001.

The decrease in TOS and the concomitant increase in TAS observed in the A-M-GelMA MN group may be associated with the regulatory effects of metformin on cellular metabolism and redox balance, as metformin has been reported in the literature to exert protective effects under oxidative stress conditions and to limit the accumulation of reactive oxygen species.^18,19^ The incorporation of amoxicillin into the formulation may have contributed to a reduction in inflammation-related oxidative stress by decreasing the microbial burden in the wound environment, and the combined use of these two agents appears to promote a more balanced cellular redox state.^60,63,64^ The lowest TOS and highest TAS values obtained in the salicylic acid-containing A-M-GelMA MN@SA group further suggest that this formulation exerts a more pronounced effect on controlling oxidative stress. Consistent with previous reports, salicylic acid at low and controlled doses have been shown to modulate cellular stress responses and to limit the oxidative burden within the wound microenvironment.^23,24^ Overall, these findings demonstrate that GelMA-based microneedle systems incorporating multiple agents can reduce oxidative stress levels while enhancing antioxidant capacity in HaCaT cells, thereby potentially creating a more favorable redox microenvironment for diabetic wound healing.

### 3.12 Cell Migration

Under diabetic conditions, impaired keratinocyte migration is a central mechanism of wound chronicity, driven by hyperglycemia-induced reactive oxygen species (ROS) accumulation that disrupts cytoskeletal organization and focal adhesion dynamics required for directed cell movement.^65,62^ The scratch assay therefore provides functional evidence of the extent to which the patch formulations can reverse or attenuate the migration deficit imposed by the 35 mM glucose model. It should also be noted that because cells were cultured in complete medium (10% FBS) without a proliferation inhibitor (e.g., mitomycin C), the measured wound closure reflects the combined contribution of keratinocyte migration and proliferation rather than migration alone.

Figure 12 shows representative phase-contrast images of the scratch wound area at 0 and 24 h for each group, along with the quantified percentage wound closure values. All hydrogel extract-treated groups demonstrated significantly greater wound closure at 24 h compared to the untreated high-glucose control (31.21 ± 5.06%), confirming that the patch extracts actively promote keratinocyte migration rather than merely preserving baseline migratory capacity under diabetic conditions. The unloaded GelMA MN achieved 55.50 ± 3.86% wound closure at 24 h, a result meaningfully higher than the control despite the absence of any pharmacological agents. This result is attributable to the biologically active nature of the GelMA matrix itself, possessing RGD adhesion sequences and providing a moist, structurally supportive microenvironment.^66^

**Figure 12:**
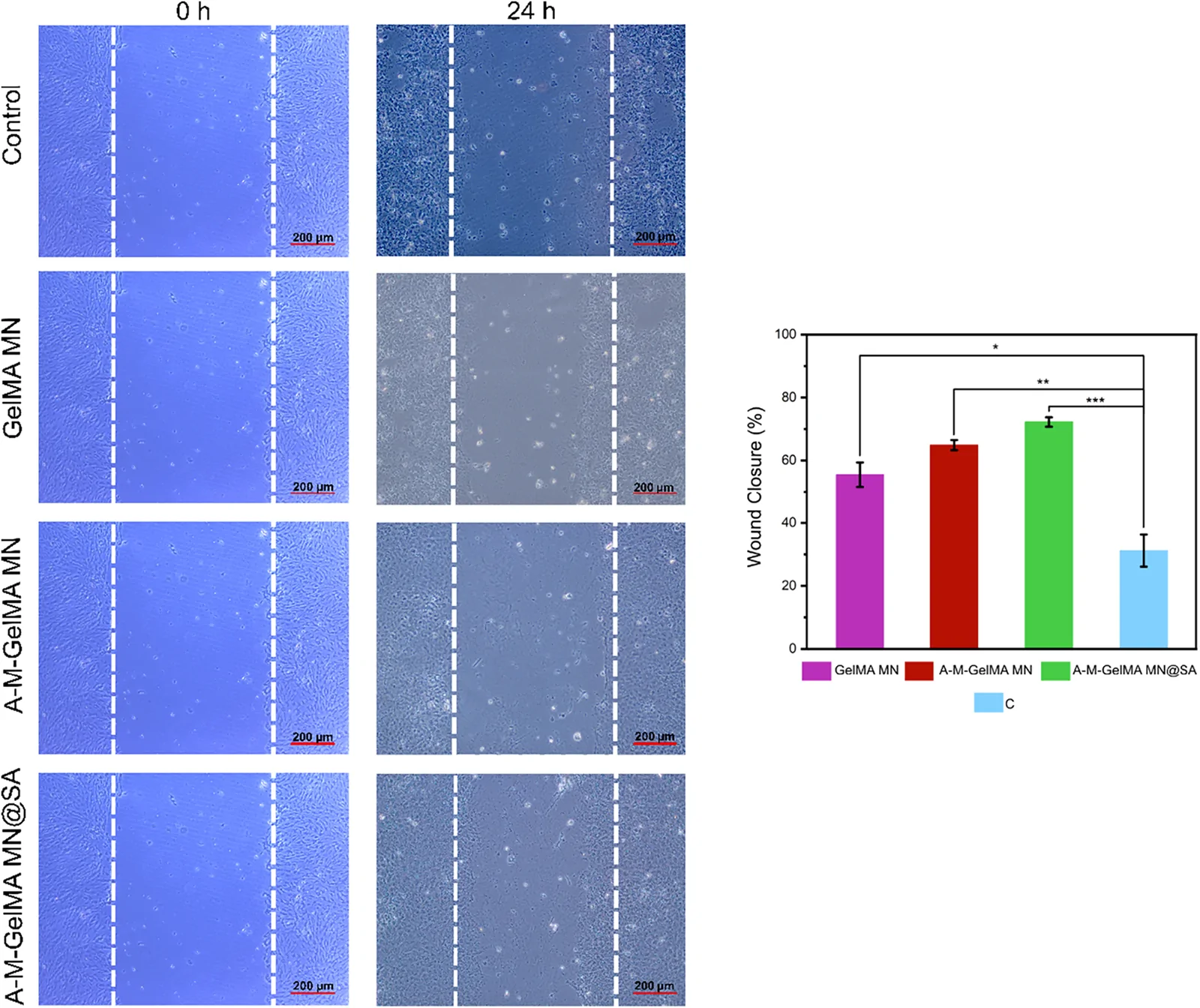
Scratch wound closure assay in HaCaT keratinocytes under simulated diabetic foot ulcer conditions, evaluated at 24 h postscratch. Representative brightfield micrographs of the scratch area for each treatment group at 0 h and 24th h (scale bar = 200 μm; images acquired at 5× magnification). Quantification of wound closure expressed as % closure relative to the initial scratch area. Data are presented as mean ± SD (*n* = 3). Statistical significance was determined by one-way ANOVA with Tukey’s post hoc test; *p < 0.05, **p < 0.01, ***p < 0.001.

Wound closure increased progressively with therapeutic complexity. A-M-GelMA MN achieved 64.90 ± 1.62%, representing a further improvement over GelMA MN attributable to the combined pharmacological contributions of amoxicillin and metformin. Metformin has been reported to modulate AMPK/mTOR signaling in keratinocytes, although the reported effects on proliferation and migration are not uniformly favorable: AMPK activation by metformin has been shown to reduce mTORC1-dependent keratinocyte motility in vitro,^67^ while metformin exposure has also been associated with reduced keratinocyte proliferation and impaired wound healing in vivo.^68^ The improvement observed here therefore cannot be assigned to a specific metformin-driven migratory mechanism.^67^

The complete A-M-GelMA MN@SA formulation achieved the highest wound closure of 72.26 ± 1.48%, representing a 41.1 percentage point improvement over the hyperglycemic control. The additional increment over A-M-GelMA MN provided by salicylic acid is mechanistically consistent with the TAS/TOS/OSI data. ROS generated under hyperglycemic conditions directly inhibit keratinocyte migration by oxidizing and inactivating the Rho-family GTPases (Rac1, RhoA) that regulate the actin remodeling required for scratch wound closure.^69^ The antioxidant activity of SA’s phenolic hydroxyl group scavenges these ROS and preserves the redox-sensitive migration. The trilayer construct produces a functional migration-promoting outcome that exceeds the capacity of any individual component.

### 3.13 Penetration of the Microneedles

Penetration analysis was conducted on parafilm for all MN samples given in Figure 13. For the blank GelMA-MN group, penetration was observed in the first three layers with 100% of MNs successfully forming holes. In the fourth layer, the penetration efficiency dropped markedly to 39.81% ± 5.78.

**Figure 13:**
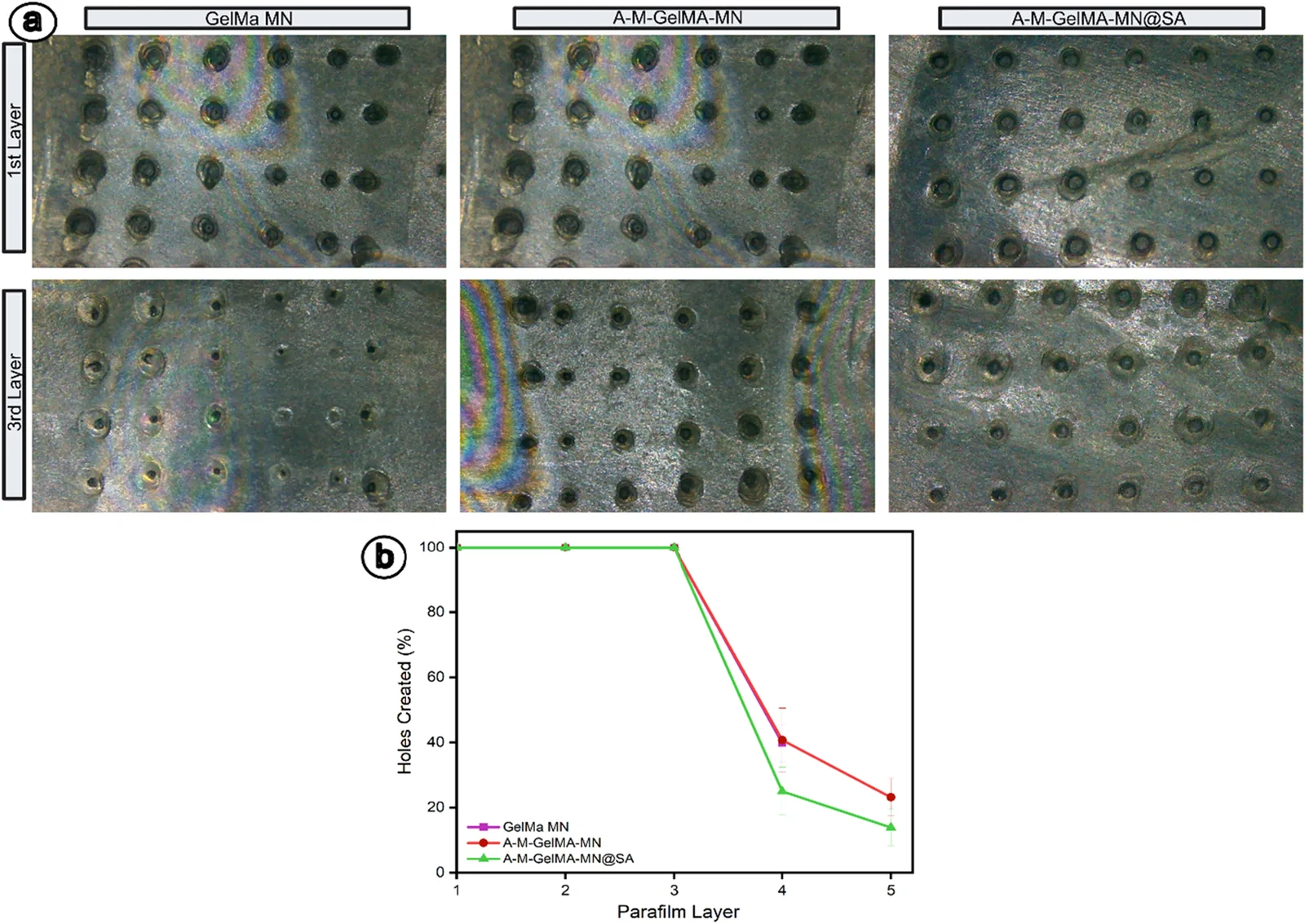
Penetration of the parafilm layers of the produced MNs (a), graph of holes created (%) to parafilm layer (b).

In the A-M-GelMA-MN group, penetration was again observed as 100% in the first three layers. The average penetration rates for the fourth and fifth layers were 40.74% ± 9.76 and 23.15% ± 5.78, respectively, corresponding to a maximum penetration depth of approximately 625 μm. This value accounts for approximately 100% of the average needle height (624.37 ± 5.55 μm).

For the A-M-GelMA-MN@SA group, penetration was complete in the first two layers, but the efficiency decreased in the subsequent layers. The third layer exhibited a penetration rate of 25% ± 7.34, the fourth layer 13.88% ± 5.55, and no penetration was observed beyond the fifth layer. Compared to the A-M-GelMA-MN group, the presence of PLA/SA microparticles appeared to reduce penetration efficiency, highlighting the influence of material composition and fabrication methods on structural performance.

In summary, all groups exhibited sufficient penetration capacity in this preliminary parafilm model, supporting further evaluation in skin or skin-mimetic substrates, with the A-M-GelMA-MN group achieving the greatest penetration depth and consistency. Compression testing indicated a slight reduction in bulk mechanical strength upon amoxicillin loading; however, this did not translate into reduced penetration performance. Penetration efficiency depends not only on compressive strength but also on needle tip geometry, microscale matrix rigidity, and the uniformity of drug distribution within the GelMA network. Metformin loading, which maintained compressive strength comparable to unloaded GelMA-MN, likely contributed to the structural performance of the A-M-GelMA-MN system during insertion.^35^

## 4 Conclusion

The present study developed and evaluated a multilayer GelMA-based microneedle platform designed for diabetic wound management. By incorporating amoxicillin, metformin, and SA into different scaffold layers, the system successfully combined antibacterial and antioxidant functions with a metformin-containing layer intended to support cellular responses under hyperglycemic conditions. Characterization analyses confirmed the structural integrity of the MNs. Drug-release studies showed biphasic release behavior over 72 h, with an initial rapid-release phase followed by a slower release phase. Mechanical testing of cylindrical samples showed adequate structural strength, and the penetration assay demonstrated insertion capacity in a preliminary parafilm model.

*In vitro* results further demonstrated that the scaffold exhibited favorable biocompatibility, with cell viability ranging from 77.98 to 95% across the tested formulations and cell models. The formulations maintained antibacterial activity against both *E. coli* and *S. aureus*, with inhibition zones reaching 22 ± 1.5 and 23 ± 0.5 mm, respectively. Degradation and swelling studies confirmed the biodegradable and hydrophilic nature of the system. Under simulated hyperglycemic conditions, the drug-loaded formulations exhibited reduced TOS levels and increased TAS values. The complete formulation also produced greater wound-gap closure than the untreated control at 24 h (72.26 ± 1.48% vs 31.21 ± 5.06%). Because the scratch assay was performed in the presence of serum and without a proliferation inhibitor, this result reflects the combined contributions of keratinocyte migration and proliferation rather than migration alone.

This study has several limitations. First, the evaluation was limited to *in vitro* assays and a parafilm penetration model. Important aspects of the diabetic foot ulcer microenvironment, including inflammation, angiogenesis, collagen deposition, vascular remodeling, macrophage polarization, and tissue regeneration, were not examined and will require *in vivo* investigation. Second, although the high-glucose culture model is widely used, it does not fully reflect other key features of diabetic foot ulcers, such as hypoxia, chronic infection, impaired vascularization, and complex immune-cell interactions. Third, the drug-release profiles were obtained from single-drug-loaded model formulations rather than from the complete multilayer construct. Taken together, these limitations highlight the need for future *in vivo* studies to evaluate the wound-healing potential and translational relevance of the platform.

Collectively, these findings support the biological plausibility and multifunctional design rationale of the microneedle platform as a candidate strategy for diabetic wound management. However, the results should be interpreted as *in vitro* proof-of-concept evidence rather than a demonstration of wound-healing efficacy. Further studies using *ex vivo* skin and *in vivo* diabetic wound models will be required to confirm the translational potential and clinical relevance of the platform.

## Supplementary Material





## Data Availability

The data sets generated during and/or analyzed during the current study are available from the corresponding author on reasonable request.

## Notes

Detailed methodology for determining drug encapsulation efficiency and drug loading; drug calibration curves (Figure S1); quantitative drug incorporation parameters (Table S1); and mathematical modeling of in vitro drug release kinetics (Figure S2 and Table S2) (PDF)
3D model of the microneedle design (GelMA MN.stl) (STL) (ZIP)
