# Supplementary material for: 3D-Printed GelMA Microneedles with Multilayer Therapeutic Properties for Diabetic Wound-Relevant Drug Delivery
Source: ACS Omega. 2026 Oct 15;11(42):64986–5003. doi: 10.1021/acsomega.6c06574 (PMC13717756; doi:10.1021/acsomega.6c06574)
Supplement: Supplementary file 1 [file ao6c06574_si_001.pdf]

Supporting Information:

3D-Printed GelMA Microneedles with Multilayer Therapeutic Properties for Diabetic Wound-Relevant Drug Delivery

Shhd Saraj[1*], **Kaan Danis**[1,2,*], Selcen Ari Yuka[1,2], Pelin Pelit Arayici[1,2], Sule Ilgar[1,2], Meryem Akkurt Yıldırım[2], Elif Kaya[3], Elif Ilhan[5], Gulgun Tınaz[4], Azime Erarslan[1], Oguzhan Gunduz[5], Cem Bulent Ustundag[1,2]

[*]These authors contributed equally to this work.

Corresponding author: Cem Bulent Ustundag (cbustun@yildiz.edu.tr)

[1]Department of Bioengineering, Faculty of Chemistry and Metallurgy, Yildiz Technical University, Istanbul 34220, Türkiye
[2]Health Biotechnology Center for Excellence Joint Practice and Research (SABIOTEK), Yildiz Technical University, Istanbul 34220, Türkiye
[3]Institute of Health Sciences, Faculty of Pharmacy, Department of Biochemistry, Marmara University, Istanbul 34854, Turkey
[4]Department of Basic Pharmaceutical Sciences, Faculty of Pharmacy, Marmara University, Istanbul 34668, Türkiye
[5]Nanotechnology and Biomaterials Research Group, Department of Metallurgical and Materials Engineering, Faculty of Technology, Marmara University, Istanbul 34840, Turkey

Materials And Method

Determination of Drug Encapsulation Efficiency and Drug Loading

For amoxicillin-loaded base and metformin-loaded microneedles, an indirect wash-loss method was utilized to account for un-crosslinked drug leached during the post-print washing stage. Immediately after printing, the patches were weighed to determine their theoretical drug content based on the initial bioink formulation. The patches were then washed in 5 ml of distilled water. The wash solutions were collected, and the concentration of the lost drug was quantified using a UV-Vis spectrophotometer (Shimadzu, Japan) at 228 nm for amoxicillin and 232 nm for metformin.

For the electrosprayed PLA microparticles, a direct extraction method was employed. A known mass of the fabricated PLA microparticles was completely dissolved in 5 mL of chloroform to release the entrapped salicylic acid. The concentration of the encapsulated drug was subsequently determined by measuring the absorbance at 298 nm.

All drug concentrations were derived from pre-established linear calibration curves. The EE% and DL% were calculated using the following equations:

For Vat photopolymerized drugs:

$$\%\mathrm{EE} = \frac{Theoretical\ Drug\ Amount - Drug\ Amount\ in\ wash}{\text{Theoretical Drug Amount}} \times 100$$

For the PLA microparticles:

$$\%\mathrm{EE} = \frac{Actual\ Drug\ Encapsualated}{\text{Theoretical Drug Amount}} \times 100$$

For all layers, the actual drug loading was calculated as:

$$\%\mathrm{DL} = \frac{Actual\ Drug\ Encapsualated}{\text{Total Mass of the Carrier}} \times 100$$

Results and Discussion

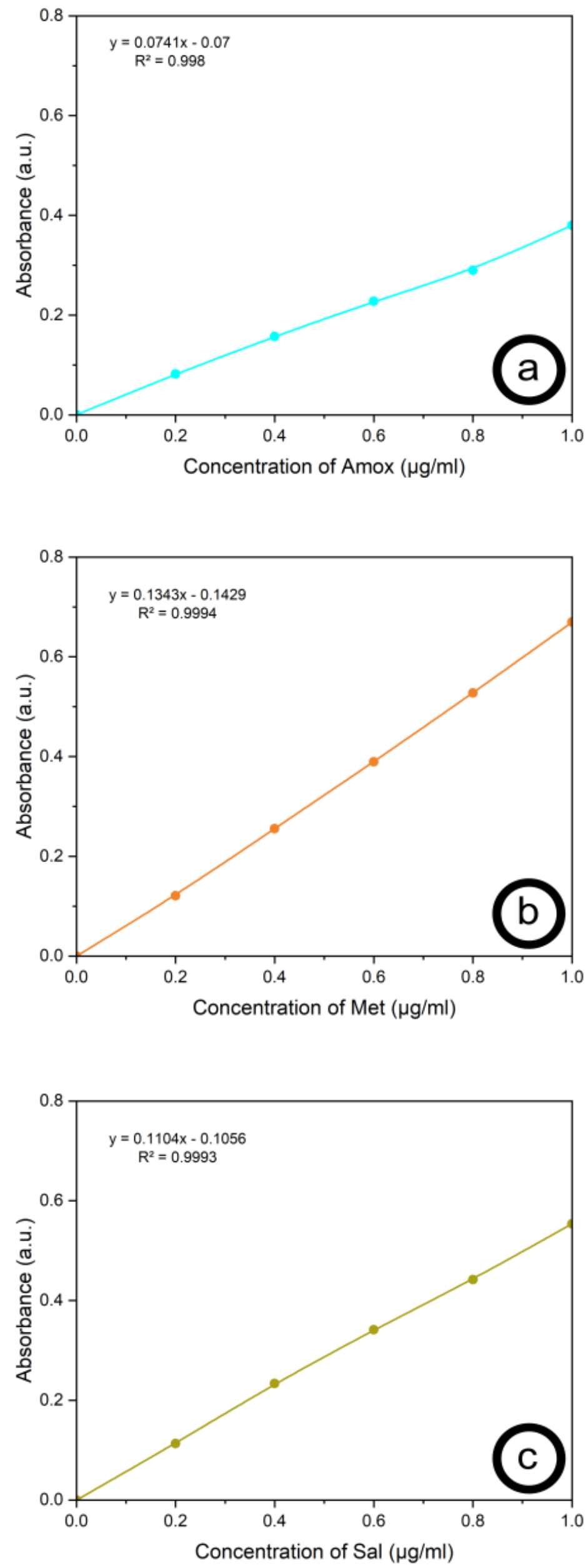

Figure S1. Calibration curves of (a) amoxicillin (λ = 228 nm), (b) metformin (λ = 232 nm) and (c) salicylic acid (λ = 298 nm)

Drug Encapsulation Efficiency and Loading Content

To confirm successful drug incorporation, dosage uniformity, and process reproducibility, the encapsulation efficiency (EE%) and actual drug loading (DL%) of the active agents were

quantified using UV-Vis spectroscopy. Because the vat photopolymerization (DLP) process directly crosslinks the drug-loaded bioink, high entrapment is theoretically achieved; however, unbound drug near the scaffold surface can be lost during the uncrosslinked-bioink washing step.

For the GelMA base (amoxicillin) and microneedle layers (metformin), an indirect wash-loss method was utilized. The post-fabrication wash solutions were collected, and the un-encapsulated drug was quantified against established calibration curves. For the electrosprayed PLA microparticles, direct matrix dissolution in chloroform was utilized to extract and quantify the encapsulated salicylic acid.

The EE% was determined to be 90.8% for amoxicillin and 76.4% for metformin. These high encapsulation values indicate minimal drug leaching during the GelMA fabrication and washing steps. The salicylic acid loaded within the PLA microparticles exhibited an EE% of 14.1%, a phenomenon characteristic of the rapid solvent evaporation dynamics inherent to electrohydrodynamic atomization (EHDA). Collectively, these quantitative data confirm that the combined DLP and EHDA fabrication methods allow for controlled and measurable dosing within the multilayer platform.

Table S1. Quantitative Drug Incorporation Parameters.

**Sample**	**Drug**	**Theoretical Loading**	**Encapsulation Efficiency (EE, %)**	**Actual Drug Loading (DL, %)**
**GelMA Base**	Amoxicillin	5.0 wt%	90.8 ± 1.2%	4.54 wt%
**GelMA MNs**	Metformin	1.0 wt%	76.4 ± 1.5%	0.76 wt%
**PLA Micro-particles**	Salicylic Acid	4.0 wt%	14.1 ± 2.1%	0.56 wt%

In Vitro Drug Release Kinetics

To better understand the physical mechanisms governing the release of amoxicillin, metformin, and salicylic acid from the 3D-printed GelMA layers and electrosprayed PLA microparticles, the in vitro cumulative release data were fitted to five standard mathematical models: Zero-order, First-order, Higuchi, Korsmeyer-Peppas, and Hixson-Crowell. The models were evaluated based on their correlation coefficients (R^2) derived from linear regression analysis.

The R^2 values for each model across the three active agents are summarized in Table S2. For amoxicillin released from the GelMA base layer and salicylic acid released from the PLA microparticles, the data exhibited the highest linearity when fitted to the Higuchi model (R^2 =

0.9595 and 0.9369, respectively), indicating that the release is predominantly controlled by diffusion through the respective matrices.

For metformin (released from GelMA MN layer), the highest correlation coefficient was observed with the Korsmeyer-Peppas model (R^2 = 0.9386), closely followed by the Higuchi model. Additionally, the release exponents (*n*) derived from the Korsmeyer-Peppas plots for all three formulations ranged from 0.70 to 0.74. The strong fit to these models, combined with the calculated release exponents, confirms that the release mechanism for these agents is primarily driven by anomalous (non-Fickian) transport, a combination of diffusion and polymer swelling typical of hydrophilic matrices, which facilitates the rapid initial therapeutic onset.

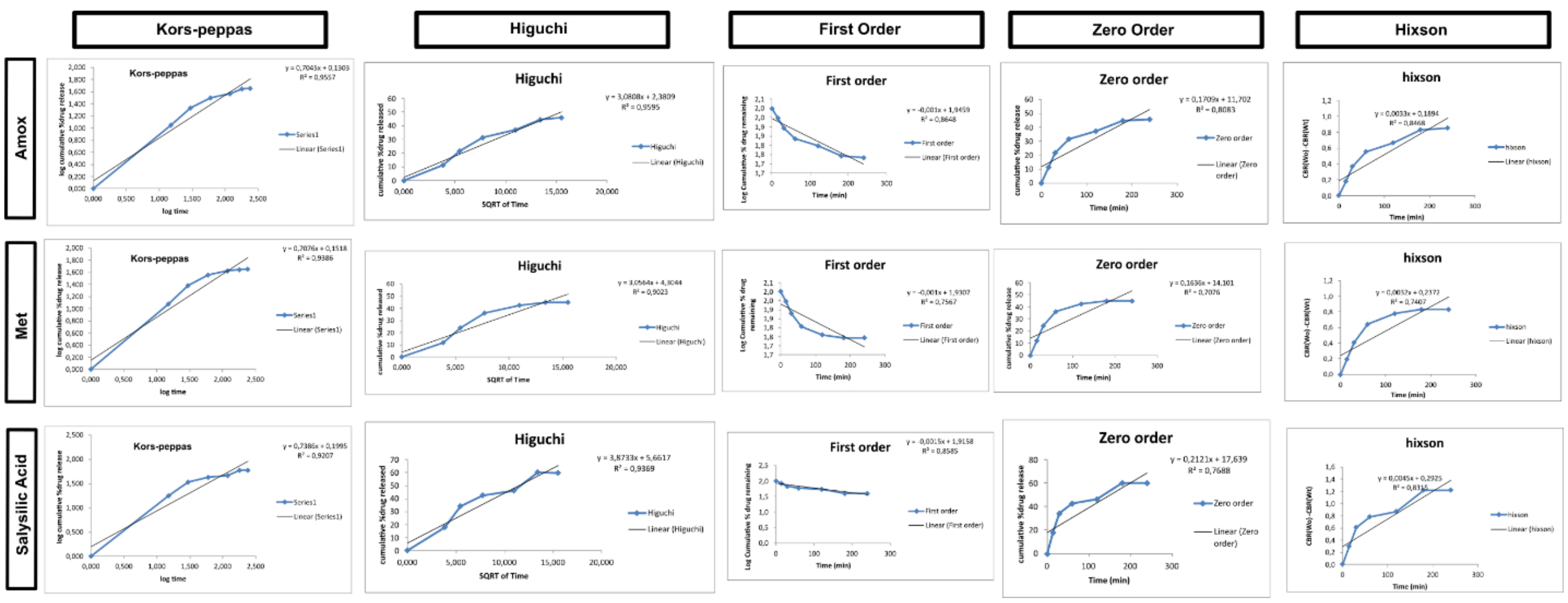

Figure S2. Release kinetics of each formulation.

Table S2. Correlation coefficients (R^2) of different mathematical models applied to the in vitro drug release data.

Drug	Zero Order	First Order	Higuchi	Korsmeyer-Peppas	Hixson-Crowell	Best Fit Model
Amoxicillin	0.8083	0.8648	0.9595	0.9557	0.8468	Higuchi
Metformin	0.7076	0.7567	0.9023	0.9386	0.7407	Korsmeyer-Peppas
Salicylic Acid	0.7688	0.8585	0.9369	0.9207	0.8315	Higuchi
